# Multilayer regulatory mechanisms control cleavage factor I proteins in filamentous fungi

**DOI:** 10.1093/nar/gku1297

**Published:** 2014-12-16

**Authors:** J. Rodríguez-Romero, M. Franceschetti, E. Bueno, A. Sesma

**Affiliations:** 1 Centre for Plant Biotechnology and Genomics (CBGP), Universidad Politécnica de Madrid, Campus de Montegancedo, 28223 Pozuelo de Alarcón, Madrid, Spain; 2 Disease & Stress Biology Department, John Innes Centre, Colney lane, Norwich NR4 7UH, UK

## Abstract

Cleavage factor I (CFI) proteins are core components of the polyadenylation machinery that can regulate several steps of mRNA life cycle, including alternative polyadenylation, splicing, export and decay. Here, we describe the regulatory mechanisms that control two fungal CFI protein classes in *Magnaporthe oryzae*: Rbp35/CfI25 complex and Hrp1. Using mutational, genetic and biochemical studies we demonstrate that cellular concentration of CFI mRNAs is a limited indicator of their protein abundance. Our results suggest that several post-transcriptional mechanisms regulate Rbp35/CfI25 complex and Hrp1 in the rice blast fungus, some of which are also conserved in other ascomycetes. With respect to Rbp35, these include C-terminal processing, RGG-dependent localization and cleavage, C-terminal autoregulatory domain and regulation by an upstream open reading frame of Rbp35-dependent TOR signalling pathway. Our proteomic analyses suggest that Rbp35 regulates the levels of proteins involved in melanin and phenylpropanoids synthesis, among others. The drastic reduction of fungal CFI proteins in carbon-starved cells suggests that the pre-mRNA processing pathway is altered. Our findings uncover broad and multilayer regulatory mechanisms controlling fungal polyadenylation factors, which have profound implications in pre-mRNA maturation. This area of research offers new avenues for fungicide design by targeting fungal-specific proteins that globally affect thousands of mRNAs.

## INTRODUCTION

The 3′-end processing of pre-mRNAs is a key step required for the formation of polyadenylated transcripts (1,2). It is a nuclear process, tightly coupled to transcription and splicing (3,4). During this process, first a specific endonucleolytic cleavage determined by CPSF73 in higher eukaryotes or Cft2/Ydh1 in yeast takes place at their 3′ ends. The 3′ UTR length varies depending at what distance from the stop codon the cleavage has occurred. This variability on 3′ UTR length is an important feature that can influence translation of mRNA isoforms (5–9). The addition of a polyadenosine tail by poly(A) polymerases completes the 3′-end processing of mRNAs. The length of poly(A) tails can influence mRNA stability, transport and/or translation, and represents an additional level of regulation (10).

Most of pre-mRNA 3′-end processing factors and polyadenylation signals of mammals and yeast have been identified and reviewed extensively (1,2,11–15). The polyadenylation machinery is a multi-subunit protein complex composed of ∼18 core proteins, although more than 80 proteins have been found to interact with pre-mRNA 3′-end processing components in humans (16). A basic metazoan 3′-end processing machinery includes the poly(A) polymerase (PAP), poly(A)-binding proteins (PABPs), the large subunits of RNA polymerase II (RNAP II) and four multi-subunits complexes: cleavage and polyadenylation specificity factor (CPSF), cleavage and stimulation factor (CstF), cleavage factor I (CFI_m_) and cleavage factor II (CFII_m_). PAP and CPSF are sufficient for both cleavage and polyadenylation reactions. The other three complexes (CstF, CFI_m_ and CFII_m_) participate in the selection of a proper poly(A) site for cleavage. PABPs regulate poly(A) tail length and activate PAP.

The poly(A) site recognition is driven by several polyadenylation signals present in the 3′-end of pre-mRNA. In mammals, distinct complexes identify these marks. By contrast, yeast poly(A) recognition signals diverge from mammalian polyadenylation signals despite sharing a tripartite poly(A) site recognition mechanism (13). Nevertheless, many yeast 3′ processing factors have counterparts in mammals, including Hrp1 for CFI_m_, CFIA (composed of Clp1, Pcf11, RNA14 and RNA15) for CstF and CFII (composed of Ydh1, Pta1, Yhh1 and Ysh1) for CPSF (15).

Typically, a canonical or more common cleavage site is present in all pre-mRNAs. However, it is frequent to find multiple potential poly(A) sites within pre-mRNAs. The selection of alternative poly(A) sites is regulated by multiple mechanisms during development and in response to environmental cues (17–19). The use of different cleavage sites within a pre-mRNA can result in presence of *cis* elements in the mRNA, or in generation of mRNA isoforms with different exon content or 3′ UTR length. The *cis* elements present in the 3′ UTRs, such as microRNA target sites, modulate gene expression by affecting cytoplasmic polyadenylation, subcellular localization, stability, translation and/or decay of the mRNA. Consequently, the selection of a proper 3′-end cleavage site represents an important regulatory step. Proteins involved in alternative polyadenylation include CFI_m_ (20,21), CstF64 (22) and Fip1 (23) in metazoans, and Hrp1 (24) in yeast.

Additional roles have been identified for human CFI_m_ and its functional orthologue Hrp1 in yeast. CFI_m_25 and CFI_m_68 possibly regulate splicing since these proteins interact with U1 snRNA 70K and U2AF 65, respectively, connecting the splicing and polyadenylation machineries (25). This is consistent with the presence of CFI_m_25 and CFI_m_68 in purified spliceosomes (26). Other roles for CFI_m_68 include its participation in 3′-end formation of animal histone mRNAs and mRNA export (27). *Saccharomyces cerevisiae* Hrp1 is an essential nucleocytoplasmic shuttling protein (28). In addition to alternative pre-mRNA processing (24), Hrp1 participates in other stages of mRNA life cycle including non-sense-mediated decay (NMD) activation (29) and mRNA export (30,31).

Compared to yeast or humans, very few studies on the polyadenylation machinery were previously carried out in filamentous fungi. Recently, we identified Rbp35 as a novel protein component of the 3′-end processing machinery present exclusively in filamentous fungi (32). Three different subdomains are present in Rbp35: one N-terminal RNA recognition motif (RRM), six Arg-Gly-Gly (RGG) tripeptide repeats and a C-terminal Met-Asp-Gly rich region. In the rice blast fungus *Magnaporthe oryzae* two Rbp35 isoforms, Rbp35A and Rbp35B, are found due to a post-translational processing at the C-terminus of the full-length protein. Rbp35A co-immunoprecipitates *in vivo* with CfI25, the orthologue of mammalian CFI_m_25, a highly conserved protein in filamentous fungi but absent in fission and budding yeasts (14). Therefore, these studies place Rbp35 as the functional equivalent of metazoan CFI_m_68, although they are not homologous proteins (32). Rbp35 is not essential for *M. oryzae* viability, and it plays a significant role as a gene-specific polyadenylation factor, regulating alternative 3′ UTR processing of infection-related mRNAs. The *Δrbp35* mutant displays significant alterations in nitrogen assimilation and grows better in the presence of rapamycin indicating a malfunction of the TOR (target of rapamycin) signalling pathway. This was corroborated using a transcriptomic approach that allowed the identification of at least five mRNA targets of Rbp35 in *M. oryzae* including *14–3–3* pre-mRNA, an important integrator of environmental cues and regulator of the TOR signalling cascade (32). These mRNA targets presented altered 3′ UTR lengths in the *Δrbp35* mutant. The yeast Hrp1 is essential for cell viability. Hrp1 orthologues are not found in metazoans or plants. Intriguingly, *M. oryzae* and other filamentous fungi have a clear orthologue of Hrp1, which suggests that combined mechanisms regulate pre-mRNA poly(A) site selection in these organisms.

In this study, we examined the regulation of *M. oryzae* Rbp35 in detail. The C-terminal processing of this protein occurs after the six RGG tripeptides. Point mutations in the six Arg influence Rbp35 localization and cleavage efficiency. Truncated forms of Rbp35 are expressed at much higher levels compared to the full-length protein, suggesting a regulatory role of the C-terminal domain of Rbp35 in maintaining its cellular concentration. Cycloheximide (CHX) treatment points out degradation signals present in the RGG region. In parallel, a proteomics approach indicates that Rbp35 also controls cellular levels of protein subsets but it is not required for general splicing or translation. Further, carbon depletion induces the transcription of two polyadenylated transcripts of ∼1000 (upstream open reading frame 1, uORF1) and ∼750 (uORF2) nucleotides (nt) in length that derive from *RBP35* 5′ UTR. The uORF1 is required for correct function of the TOR kinase pathway on minimal media. The role of two additional CFI proteins in *M. oryzae*, Hrp1 and CfI25, is further analysed to understand why filamentous fungi have maintained proteins with apparently redundant functions. Here, we confirmed their involvement in fungal development and plant pathogenicity. Interestingly, Rbp35A, Hrp1 and CfI25 cannot be detected in carbon-starved cells, suggesting that pre-mRNA processing events are affected in nutrient-poor media. In summary, our data suggest that CFI proteins exhibit different regulatory layers, which correlate with the multi-faceted strategy of *M. oryzae* to adapt to diverse environments.

## MATERIALS AND METHODS

### Fungal media, growth conditions and infection assays

Fungal strains were grown on different media composition: CM (complete medium) and MM (minimal medium) (33), and DCM (defined complex medium) (34). Fungal growth on nitrogen and carbon starvation was carried out on MM depleted of nitrogen or carbon sources. Leaf and root infections were conducted as previously described (35). All the fungal strains generated and used in this study are described in Supplementary Table S2.

### Generation and imaging of Rbp35:mRFP variants

*Magnaporthe oryzae* genes used in this study are based on the eighth annotation of the genome available at The Broad Institute (http://www.broadinstitute.org/annotation/genome/magnaporthe_comparative/MultiHome.html) and EnsemblFungi (http://fungi.ensembl.org/index.html). Rbp35 protein variants were generated using multi-site gateway technology (Life Technologies), the binary destination vector SULPH-R3R4 (32) and polymerase chain reaction (PCR) fragments amplified from either *M. oryzae* genomic DNA or cDNA (intron-less construct). PCRs were performed using Phusion DNA polymerase (NEB) and primers detailed in Supplementary Table S3. Basic molecular biology techniques, such as gel electrophoresis, cloning, restriction enzyme digestion and gel blots, were performed using standard procedures (36). Selection of fungal transformants was always carried out on DCM using the corresponding antibiotic. Quantification of Rbp35 short/long isoform ratios from western blots was carried out using the ImageJ software (http://rsb.info.nih.gov/ij/). Visualization of fungal cells containing mRFP (cherry variant) constructs was performed with a Leica TCS SP8 confocal microscope. green fluorescent protein (GFP) was excited using the 488-nm laser line from an argon ion laser, and the emission was captured using a 505–550 nm band-pass filter. mRFP (cherry) was excited using the 561-nm laser line, and the emission was captured using a 575–615 nm band-pass filter.

### Proteomic and quantitative PCR (qPCR) analyses

RNA and protein extractions were performed as previously described (32). Three biological replicates were run in 2D gels for each treatment (six gels in total). Proteins with consistently different spot intensities between the wild-type (WT) and *Δrbp35* strains were picked and digested with trypsin. Samples were identified by mass spectrometry at the Joint Proteomics Facility of the John Innes Center-Institute of Food Research (Norwich, UK). Sample peptide fingerprints obtained by MALDI-TOF analyses were searched against the *M. oryzae* genome database using the searching algorithm MASCOT Peptide Mass Fingerprint Search tool (Matrix Science, London, UK, http://www.matrixscience.com).

To compare the relative abundance of gene transcripts, the average threshold cycle (*Ct*) was normalized against actin transcript and relative quantification of gene expression was calculated by the 2^-^^ΔΔ*Ct*^ method (37). Four dilutions of all cDNA samples were used to test primer efficiency. qPCR reactions were performed using 1 μl of reverse transcribed products as template and fast-start DNA master SYBR green I kit (Roche Diagnostics) in a final reaction of 20 μl with the following program: one cycle of 95ºC for 4 min and 40 cycles of 94ºC for 30 s and 60ºC for 30 s. The amplification of the target genes was monitored every cycle by SYBR-green fluorescence. The *Ct* (threshold cycle) was used as a measure for the starting copy numbers of the target gene. The qPCR analysis was carried out using three technical repetitions from three independent biological experiments for each gene.

### Quantification of melanin and flavonoid profiles

Phenylpropanoid and melanine extraction protocols were respectively adapted from (38) and (39,40). Two hundred mg of WT and *Δrbp35* mycelium powder ground with liquid nitrogen were vortexed with 1 ml of 70% methanol thoroughly for 5 min at room temperature. Then, 200 μl of distilled water was added to each sample, vortexed for 30 s twice and centrifuged 1 min at maximum speed. Supernatants were transferred to another tube, spun down again for cleaning-up at maximum speed for 2 min and used directly for phenylpropanoid content analyses at the John Innes Centre metabolomics service. On the other hand, pellets were kept at 4ºC for analysis of their melanine content. Pellets were mixed for 30 s with 1 ml of 10% TCA by vortexing, and centrifuged to remove the supernatant. This step was repeated twice and two washes of pellets with 1 ml of 100% ethanol were performed. The final pellets were resuspended in 750 μl of 1N NaOH + 10% DMSO with the help of a spatula, mixed by vortexing for 30 s and left at 60ºC overnight. Then, these pellets were incubated at 80ºC for either 20 min or 3 h, and centrifuged 5 min at maximum speed. Supernatants were measured in a spectrophotometer at 470, 500 and 600 nm after setting the blank with 1N NaOH + 10% DMSO.

### Generation of Hrp1 and CfI25 knockouts and complemented strains

The hygromycin resistance cassette was amplified from the binary vector pCT74 (41) using the primers B1-Hygr and B2-Hygr-stop and cloned into gateway entry plasmid pDONR207. The 5′ and 3′ regions of *HRP1* and *CFI25* were amplified by PCR using the primers described in Supplementary Table S3. The amplicons were cloned into the gateway entry plasmids pDONR P4-P1R and pDONR P2R-P3. The final gateway clone was created by LR recombination reaction with these three entry clones and the binary destination vector pGKO2-R3R4 derived from pGKO2, which contains the HSVtk cassette for conditional negative selection marker against ectopic transformants (42). Final constructs were introduced into *Agrobacterium tumefaciens* AGL1. Selected bacterial colonies were used for Agrobacterium-mediated transformation in *M. oryzae* WT strain Guy11. Fungal transformants were selected using simultaneously 200 mg/ml hygromycin and 5-fluoro-2′-deoxyuridine (50 μM), and evaluated for their growth on DCM solid media. Strains able to grow under these conditions only contained a single insertion. The knockout strains were confirmed by PCR (Supplementary Figure S6). CfI25 and Hrp1 proteins tagged with GFP or HA-FLAG were also generated using multi-site gateway technology (Invitrogen). PCRs for BP recombination reactions were performed using primers detailed in Supplementary Table S3.

## RESULTS

### Proteolytic processing of *M. oryzae* Rbp35 occurs after the six RGG tripeptides

**Figure 1.**
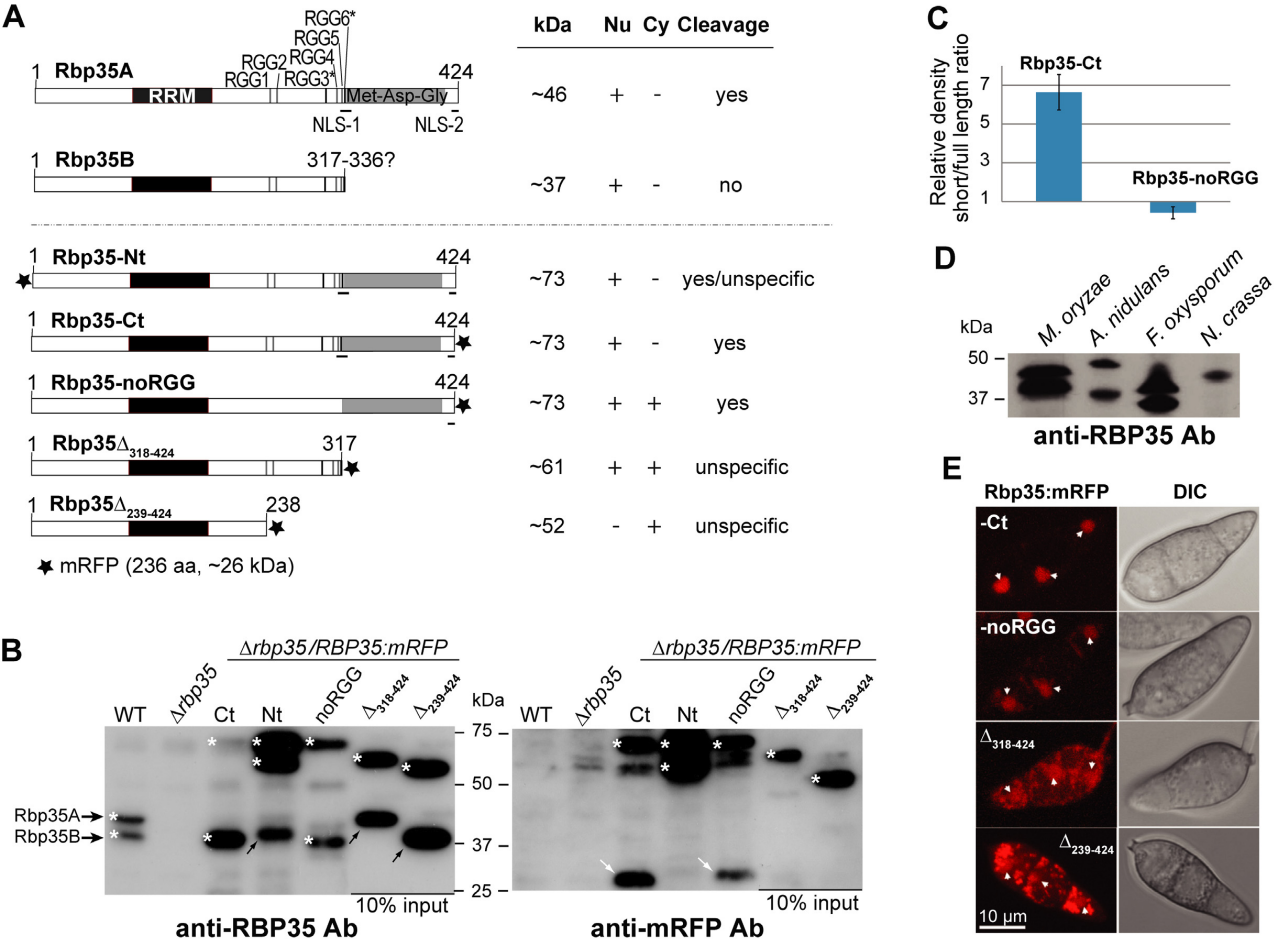
Nuclear localization and processing signals are located at the C-terminus of Rbp35. (**A**) Rbp35:mRFP constructs, including the native isoforms (Rbp35A and Rbp35B) with their predicted size (kDa), localization and proteolytic processing pattern. RGG* contains good consensus sequence for Arg methylation (F/G GGRGG G/F) (44). Table description; protein present (+) or absent (-) in nucleus (Nu) and cytoplasm (Cy); protein subject to correct cleavage (yes), incorrect cleavage (unspecific) or protein not cleaved (no). NLS, nuclear localization signal. (**B**) Immunoblots of total protein extracts derived from WT, *Δrbp35* and *Δrbp35* complemented with Rbp35:mRFP variants. Protein fragments containing mRFP are indicated by white arrows while black arrows show the by-products after unspecific Rbp35 processing. White asterisks show Rbp35 intact isoforms and derived Rbp35:mRFP variants. Unspecific processing and lack of release of the C-terminal mRFP are observed in the two truncated Rbp35:mRFP proteins (see further explanation in Supplementary Figure S1). (**C**) Quantification of protein band intensities of Rbp35A:mRFP and Rbp35B derived from Rbp35-Ct and Rbp35-noRGG using three different immunoblots and ImageJ software. In Rbp35-Ct, Rbp35B is 6-fold more abundant than Rbp35A:mRFP. By contrast, the full-length Rbp35A:mRFP is slightly more abundant than Rbp35B in Rbp35-noRGG. (**D**) Rbp35 processing is also observed in the strains of *A. nidulans* FGSC A4 and *F. oxysporum* f.sp. *lycopersici* 4287. Rbp35 is not cleaved in *N. crassa* FGSC 2489 despite this fungal species is taxonomically closer to *M. oryzae*. (**E**) Confocal images of *Δrbp35* conidia complemented with different *Rbp35:mRFP* constructs. Nuclei are indicated by white arrowheads. DIC, differential interference contrast images. Full-length mRFP fusion construct (Rbp35-Ct) shows a nuclear localization as previously described. Mutations in the RGG module and truncations in Rbp35 C-terminal end contribute to its mislocalization.

Rbp35 undergoes a proteolytic cleavage at the C-terminal end of the protein, generating two Rbp35 isoforms in the cell: the full-length protein (Rbp35A) and a smaller isoform (Rbp35B) (32) (Figure 1A and B and Supplementary Figure S1). We set out first to construct several C-terminally tagged mRFP variants of Rbp35 to identify the cleavage site and additional regulatory subdomains within the protein. Two C-terminal truncations of Rbp35:mRFP, lacking either the Met-Asn-Gly region (Rbp35Δ_318–424_) or both the RGGs and Met-Asn-Gly modules (Rbp35Δ_239–424_), were generated for further functional analysis (Figure 1A and Supplementary Figure S2A). It has been shown that the methylation status of the RGG tripeptide regulates the subcellular location of RNA-binding proteins (43). Consequently, the RGG module of Rbp35, which contains six RGG tripeptides with two potential consensus sequences for Arg methylation (44) (Figure 1A), was also modified by mutating all the six Arg. This additional protein variant, tagged at the C-terminus with mRFP, was named Rbp35-noRGG (Figure 1A, Supplementary Figures S1 and S2A).

The three mutated Rbp35:mRFP constructs were reintroduced into *Δrbp35* and their processing pattern was examined by western blotting (Figure 1B and Supplementary Figure S1). As a control, previously generated full-length translational fusions of mRFP in the N- and C-terminus of Rbp35 were used, Rbp35-Nt and Rbp35-Ct, respectively (32). The short isoform Rbp35B generated from Rbp35-noRGG indicated that the cleavage site was not located at the Arg of the RGG cluster although the efficiency of the cleavage was clearly affected in Rbp35-noRGG as shown by the quantification of the protein bands detected by western blotting (Figure 1C). The failure of Rbp35Δ_318–424_ and Rbp35Δ_239–424_ to generate a small band of mRFP revealed that the proteolytic processing of Rbp35 occurred after the last RGG tripeptide. We observed that the mRFP tag generated additional cleavage sites in the Rbp35:mRFP variants (Supplementary Figure S1). Peptidic fragments of variable sizes not recognized by the anti-mRFP antibody were detected in Rbp35Δ_318–424_ and Rbp35Δ_239–424_ with the anti-Rbp35 antibody, suggesting that Rbp35Δ_318–424_ and Rbp35Δ_239–424_ proteins underwent unspecific cleavage (black arrows, Figure 1B and Supplementary Figure S1). The full-length amino and carboxy fusions Rbp35-Nt and Rbp35-Ct were correctly cleaved. However, Rbp35-Nt generated an additional unspecific fragment not recognized by the anti-mRFP antibody (black arrow, Figure 1B), and the expected mRFP-containing fragment of Rbp35-Ct is smaller (Supplementary Figure S1). Possibly, the mRFP tag affected differently and introduced new processing sites in Rbp35:mRFP variants. Surprisingly, the protein abundance of Rbp35Δ_318–424_ and Rbp35Δ_239–424_ was ∼10 times higher than the full-length mRFP protein fusions (only 10% input from total protein extracts were used in western blots; Figure 1B).

We investigated if the processing observed in *M. oryzae* Rbp35 was conserved in other fungal species. *Aspergillus nidulans*, *Fusarium oxysporum* and *Neurospora crassa* isolates, whose genome sequences are available, contain a clear orthologue of *M. oryzae* Rbp35 (Supplementary Figure S2B). Rbp35-specific bands were detected in each fungal isolate by western blot using the anti-Rbp35 antibody raised against the entire protein of *M. oryzae* Rbp35 (Figure 1D). Rbp35 proteins of *A. nidulans* and *F. oxysporum* showed two isoforms of the protein, suggesting that Rbp35 is also processed in these fungal species. Despite *N. crassa* is a close relative of *M. oryzae*, a single band was visible in this fungal species, which indicated that either this fungus possesses a unique isoform of Rbp35 or that the proteolytic cleavage occurs in different growth conditions.

Accordingly, we conclude that (i) the C-terminal processing of *M. oryzae* Rbp35 occurs after the RGG cluster, (ii) the six arginines within the RGG domain and their potential methylation influence the efficiency of the cleavage, (iii) the Met-Asn-Gly region autoregulates its protein levels in *M. oryzae* and (iv) Rbp35 processing is conserved in some Pezizomycotina fungal species. *Aspergillus* and *Fusarium* genera include plant and human pathogenic species while *Neurospora* species are saprophytes. Consequently, a correlation exists between fungal pathogenicity and Rbp35 processing among the studied strains.

### RGGs and Met-Asn-Gly domain regulate Rbp35 localization and function

Previous studies showed that both Rbp35 isoforms present a steady-state nuclear localization pattern in *M. oryzae* (32). We examined the contribution of the Arg from the RGG module and C-terminal domains in the nuclear localization of Rbp35. Rbp35-noRGG was visualized in conidial nuclei (Figure 1E). However, a diffuse signal was clearly observed in the cytoplasm surrounding the nuclei, which suggests that the six Arg of the RGG domain may contribute to its nuclear import (Figure 1E). The Rbp35Δ_318–424_ protein variant was present in the nucleus despite lacking one of the two bipartite nuclear localization signal (NLS-1 and NLS-2) present in the full-length protein (Figure 1A and Supplementary Figure S2A). This indicates that either NLS-1 itself can drive Rbp35Δ_318–424_ to the nucleus or Rbp35Δ_318–424_ may interact with other nuclear-localized proteins. By contrast, Rbp35Δ_239–424_ was mainly observed on cytoplasmic foci and excluded from nuclei. The strong fluorescence signal of Rbp35Δ_318–424_ and Rbp35Δ_239–424_ compared to the full-length Rbp35:mRFP (Rbp35-C) correlated with their high expression level within the cell (Figure 1B). Highly fluorescent granules were observed in the spores of Rbp35Δ_318–424_ and especially in Rbp35Δ_239–424_ spores_._ This pattern is possibly an artefact associated to their overexpression.

**Figure 2.**
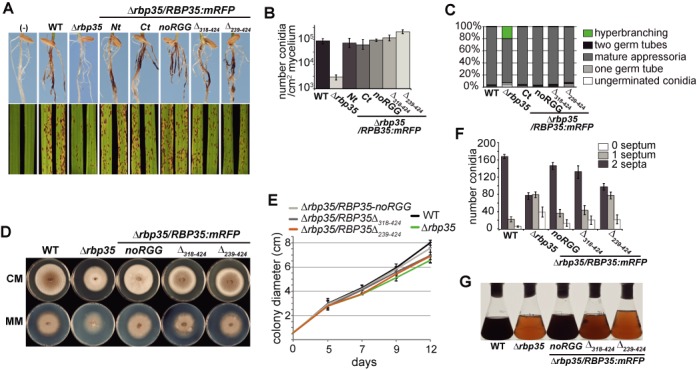
Rbp35:mRFP variants rescue partially or completely *Δrbp35* defects. (**A**) Rbp35:mRFP variants restore *Δrbp35* virulence deficiencies on leaves and roots except for Rbp35Δ_239–424_. The *Δrbp35*/Rbp35Δ_239–424_ strain shows an intermediate phenotype on leaves. (**B** and **C**) Rbp35:mRFP variants fully restore *Δrbp35* conidia production and hyperbranching defects. The *Δrbp35*/Rbp35Δ_239–424_ produces 2-fold more conidia than the WT strain. Values represent mean of three experiments. (**D** and **E**) Colony morphology on CM or MM, and growth curves on CM of WT, *Δrbp35* and *Δrbp35* complemented with Rbp35:mRFP variants. *Δrbp35*/Rbp35Δ_318–424_ and *Δrbp35*/Rbp35Δ_239–424_ show partial recovery. *Δrbp35*/Rbp35-noRGG can grow almost like the WT strain. (**F**) Rbp35-noRGG, Rbp35Δ_239–424_ and Rbp35Δ_239–424_ partially restore the *Δrbp35* deficiencies in conidial septum formation (mean ± SD of three biological replica). (**G**) Culture filtrates of WT and *Δrbp35* strains after 48 h on liquid CM. *Δrbp35* strains containing either Rbp35Δ_318–424_ or Rbp35Δ_239–424_ cannot restore the defects of *Δrbp35* in the synthesis of pigmented metabolites.

Next, we tested the ability of the three Rbp35:mRFP variants to rescue the *Δrbp35* mutant phenotype. Rbp35Δ_239–424_ and Rbp35Δ_318–424_ restored full disease symptoms on roots, production of conidia and hyperbranching growth on coverslips (Figure 2A–C). On leaves, the *Δrbp35/*Rbp35Δ_239–424_ strain showed intermediate phenotype while *Δrbp35/*Rbp35Δ_318–424_ produced WT symptoms.The *Δrbp35* strains containing the two truncated and Rbp35-noRGG constructs presented improved growth rates and conidial septum formation compared to *Δrbp35* but were not restored to WT levels (Figure 2D–F). In addition, *Δrbp35/*Rbp35Δ_239–424_ and *Δrbp35/*Rbp35Δ_318–424_ were clearly deficient in the production of secreted pigmented compounds (Figure 2G). The fact that Rbp35-noRGG rescued all the deficiencies observed in *Δrbp35* mutant except for subtle differences in growth and septum formation, points out that Arg substitutions in the RGG cluster did not significantly affect the function of Rbp35 under the conditions tested.

Relaying on protein size and mass spectrometry, we know that the short isoform Rbp35B includes at least four of the six RGG tripeptides (32). The truncated protein Rbp35Δ_318–424_, which contains an intact RGG region and gives a protein band size similar to Rbp35B:mRFP cannot fully recover Δ*rbp35* defects. This suggests that the Met-Asn-Gly domain is important for Rbp35 function, and that Rbp35A, the full-length isoform of Rbp35 has a functional role in *M. oryzae*.

### The Met-Asn-Gly domain tightly regulates cellular concentrations of Rbp35

**Figure 3.**
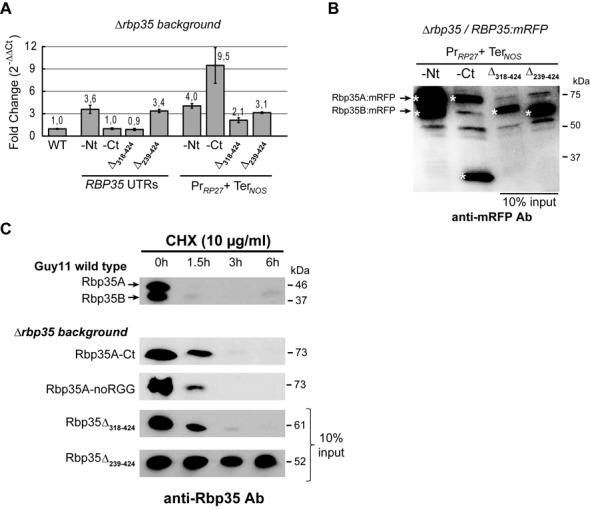
The Met-Asn-Gly and RGG regions of Rbp35 regulate protein synthesis and degradation rates. (**A** and **B**) Expression of *Rbp35:mRFP* constructs driven by either *RBP35* UTRs or *RP27* promoter and *NOS* terminator in *Δrbp35* background. (**A**) Analysis by qPCR of *RBP35* mRNA levels in WT and *Δrbp35* strains complemented with *Rbp35:mRFP* variants. Expression of *Rbp35:mRFP* constructs driven by the *RP27* promoter is clearly higher. However, no correlation exists between mRNA and protein levels among these constructs. (**B**) Immunoblots of Rbp35:mRFP proteins under the control of *RP27* promoter and *NOS* terminator. Rbp35Δ_318–424_ and Rbp35Δ_239–424_ still present 10-fold increased expression, indicating that *RBP35* UTRs are not involved in this overexpression effect. White asterisks show Rbp35:mRFP variants and the C-terminally processed mRFP. (**C**) Western blot analysis after CHX treatment highlights the presence of degradation signals in the RGG-containing region of Rbp35. Only the protein variant Rbp35Δ_239–424_ that lacks the RGG domain remains stable in the cell.

We decided to examine why the proteins Rbp35Δ_239–424_ and Rbp35Δ_318–424_ were expressed at much higher levels than the N- and C-terminal mRFP fusions of the full-length protein, Rbp35-Nt and Rbp35-Ct, respectively. It is worth noting that all these constructs were generated using *RBP35* native 5′ and 3′ UTRs. We performed qPCRs of the Δ*rbp35* strains containing the Rbp35:mRFP variants. Similar amounts of transcripts were observed between *RBP35-Nt* and *RBP35Δ_239–424_* constructs (Figure 3A). By contrast, western experiments revealed that Rbp35Δ_239–424_ is ∼10 times more abundant than Rbp35-Nt (Figure 1B), indicating a lack of correlation between mRNA and protein levels for this truncated variant. The same was true for Rbp35-Ct and Rbp35Δ_318–424_. These two constructs produced similar transcripts levels but Rbp35Δ_318–424_ protein was about 10 times more abundant than Rbp35-Ct (Figures 1B and 3A). Furthermore, Rbp35-Ct always showed lower signal intensity by western blot analysis compared to Rbp35-Nt, which could be explained by the 3-fold difference in transcripts levels among these two full-length constructs (Figures 1B and 3A and B).

To investigate further the high cellular concentration of Rbp35Δ_239–424_ and Rbp35Δ_318–424_, the native 5′ and 3′ UTRs of *RBP35* were replaced by the strong promoter of the ribosomal protein *RP27* (45), and the terminator of the nopaline synthase gene (*NOS*) in full-length and truncated mRFP constructs of *RBP35*. The effect of the strong promoter was clear: all these constructs showed equal or increased mRNA levels compared to the corresponding constructs with native *RBP35* UTRs (Figure 3A), although protein rates among different variants remained similar (Figure 3B). Pr*_RP27_*:*RBP35Δ_318–424_*:Ter*_NOS_* and Pr*_RP27_*:*RBP35Δ_239–424_*:Ter*_NOS_* continued producing ∼10 times more protein than the full-length constructs, even if the Pr*_RP27_*:*RBP35-Ct*:Ter*_NOS_* produced higher mRNA levels.

The elevated protein expression of Rbp35Δ_239–424_ and Rbp35Δ_318–424_ suggests that the Met-Asn-Gly domain is required to adjust cellular concentrations of Rbp35. This domain may be interacting with protein components of the translation machinery to regulate its own translation, i.e. absence of this domain in Rbp35Δ_239–424_ and Rbp35Δ_318–424_ triggers a response to increase their translation rates. Alternatively, the Met-Asn-Gly domain could be targeted by the proteasome or cellular proteases, acting as a sensor of Rbp35 intracellular levels. We concluded that the C-terminal region of Rbp35 acts as an autoregulatory domain of Rbp35 since truncated variants lacking this region increase ∼10-fold their cellular concentration. Moreover, *RBP35* 5′ and 3′ UTRs are not involved in the control of Rbp35 protein levels in the fungal cell.

### The RGG region targets Rbp35 for degradation

To further assess if the regulation of Rbp35 cellular concentration is at the level of translation or protein stability, WT and *Δrbp35* strains containing different truncated variants were treated with CHX, which inhibits global protein synthesis (Figure 3C). After 90 min treatment with CHX, neither of the two isoforms were found in the cell, indicating that Rbp35 undergoes a rapid turnover in the cell. The Rbp35:mRFP variants Rbp35-Ct, Rbp35-noRGGs and Rbp35Δ_314–424_ showed slightly more stable pattern compared to the WT full-length isoform Rbp35A. After 6 h of CHX treatment only the truncated variant Rbp35Δ_239–424_ was detected in fungal cells, which suggested that degradation signals are present in the RGG-containing region. Since Rbp35Δ_318–424_ remains 10-fold up-regulated despite it is being degraded, these results suggest that the Met-Asn-Gly domain plays an autoregulatory role regulating Rbp35 translation.

### Rbp35 regulates cellular levels of a subset of proteins

**Figure 4.**
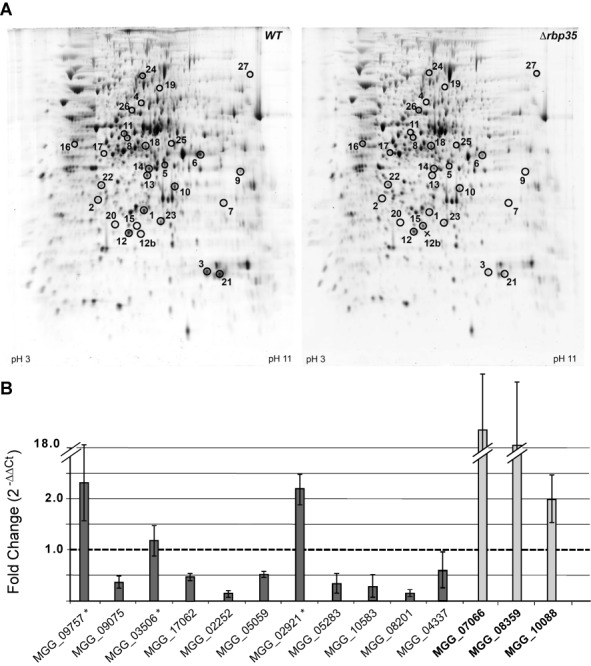
Rbp35 can regulate protein levels. (**A**) 2D electrophoresis gels of total protein extracts from WT (left) and *Δrbp35* (right). Correlation between the spot numbers and *M. oryzae* proteins are indicated in Supplementary Table S1. Enzymes involved in melanin and flavonoid biosynthesis are affected in *Δrbp35*. (**B**) Expression analysis of down- and up-regulated (bold) genes in *Δrbp35* reveals a direct correlation between their mRNA and protein levels except for MGG_02921, MGG_09757 and MGG_03506 (marked with an asterisk).

Since our results suggested that Rbp35 could regulate its own translation, we carried out a comparative proteomics approach using the WT strain and the Δ*rbp35* mutant to investigate if the lack of Rbp35 was causing significant changes in other cellular proteins. We performed 2D electrophoresis gels of total protein extracts from WT and *Δrbp35* strains and compared protein expression profiles of these two strains. We consistently identified 20 proteins down-regulated and 7 up-regulated in *Δrbp35* (Figure 4A, Supplementary Table S1). Remarkably, 18 (67%) of these proteins contained Interpro motifs associated with primary and secondary metabolism, correlating with the defects in secreted natural products observed in *Δrbp35* (Figure 2G). In particular, enzymes required for melanin synthesis such as the scytalone dehydratase (SDH; MGG_05059) and the tetrahydroxynaphthalene reductase (3THNR; MGG_02252) were over 2-fold down-regulated, suggesting alterations in the melanin synthesis pathway. In addition, two proteins (MGG_06917; MGG_10583) linked to flavonoid metabolism were respectively 3.0- and 2.7-fold down-regulated. According to protein domains and well-characterized orthologues in yeast, the nine remaining proteins were involved in cell wall integrity (MGG_09757), mitochondrial function (MGG_07066; MGG_07752; MGG_09075), signalling (MGG_08622), mRNA stability/degradation (MGG_03506), oxidation/reduction of thiol groups (MGG_017602), stress response (MgKATG1; MGG_04337) and heterochromatin silencing (MGG_15048). To confirm some of our proteomics results, we carried out a quantification of melanin extracts in WT and *Δrbp35* strains, and used an high pressure liquid chromatography (HPLC) profile of phenylpropanoids as a simple measure of diversity. A 35–50% reduction on melanin content was detected by colorimetric assays and the HPLC profile of phenylpropanoids was also altered in *Δrbp35* (Supplementary Figure S3). Such a reduction suggest a link between protein levels of these metabolic enzymes and the cellular content of melanins and phenylpropanoids in the *Δrbp35* mutant. To study the correlation between the 27 up- and down-regulated proteins in the mutant and their transcript levels, we looked first at the comparative transcriptome profile of *Δrbp35* carried out under the same growth conditions (32). Seven out of the 20 down-regulated proteins showed no changes or increased mRNA levels in the microarray experiment (Supplementary Table S1). We selected 14 genes including these seven genes for qPCR analysis and found that three of them (MGG_02921, MGG_09757 and MGG_03506) showed an inverse correlation between protein and mRNA levels (Figure 4B). Accordingly, either these three proteins were not being translated properly in the mutant background, or they were being degraded more rapidly. The rest of the up- and down-regulated transcripts analysed by qPCR showed a direct correlation with their protein content in *Δrbp35*, i.e. up-regulated proteins displayed higher mRNA levels and, similarly, down-regulated proteins displayed lower mRNA levels.

We assumed that the high protein conservation pattern between WT and Δ*rbp35* strains excluded Rbp35 from contributing to global regulation of splicing or translation. Interestingly, three down-regulated proteins in Δ*rbp35* presented WT or up-regulated mRNA levels, which suggest an involvement of Rbp35 in the regulation of their mRNA translation or protein degradation. This effect might be due either to a direct involvement of Rbp35 with the translation machinery or to its role in alternative polyadenylation, since mRNA isoforms can be translated with different efficiencies.

### Concomitant reduction of Rbp35A protein levels and induction of 5′ UTR polyadenylated transcripts in starved cells

**Figure 5.**
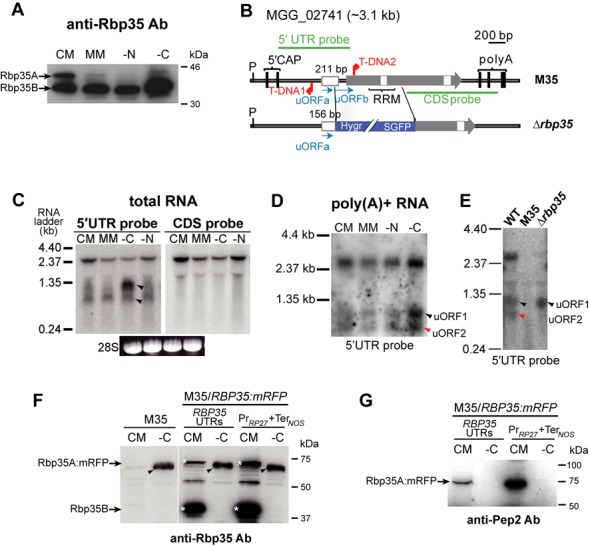
Regulatory features of Rbp35. (**A**) Western blot of total proteins extracts under different nutrient conditions showing a controlled proteolytic degradation of Rbp35. The Rbp35A isoform is hardly detected in low nutrient content media. (**B**) Diagram of the *RBP35* locus in the T-DNA mutant M35 and in the knockout strain *Δrbp35*. M35 contains two tandem T-DNA insertions, one within the 5′ UTR and the second after the ATG of the ORF. By contrast, the *Δrbp35* mutant lacks a 1.0-kb region of the gene although still maintains the majority of the 5′ UTR intron. (**C**) Two additional RNAs of ∼0.75 and ∼1.0 kb indicated with black arrowheads are produced from the 5′ UTR of *RBP35*. (**D**) These RNAs are polyadenylated and can be detected using a single-stranded antisense 5′ UTR probe. (**E**) These two RNAs (arrowheads) are not found in the 5′ UTR of the T-DNA mutant M35 while only the 1 kb transcript is detectable in *Δrbp35*. (**F**) M35 produces a protein (arrowhead) that is recognized by the anti-Rbp35 antibody in carbon-depleted cells. White asterisks show Rbp35A:mRFP and Rbp35B. (**G**) This protein cannot be detected with an antibody designed against the N-terminus of Rbp35 (anti-Pep2).

To analyse if the two Rbp35 isoforms are always present in the cell, we carried out a western blot analysis of total protein extracts derived from fungal mycelia grown on CM and MM. It included nitrogen (MM-N) and carbon (MM-C) depletion. Strikingly, the full-length isoform Rbp35A was hardly detected by western blotting when fungal cells were grown on MM, or MM-N and MM-C for 12 h (Figure 5A). This result suggested that Rbp35A degradation and/or reduction of its translation rate was programmed by the fungal cell to overcome environmental stress.

The *RBP35* gene locus has a potential uORFa 111 bp long within its 211 bp 5′ UTR intron (Figure 5B and Supplementary Figure S4A and B). This small ORF can encode a 36 aa peptide. Three additional ATGs in frame are present within the intron, and are located after the stop codon of uORFa (Supplementary Figure S4B). The first ATG generates a second uORF of 216 bp in length (uORFb; Supplementary Figure S4A and B). The uORFb expands 26 bp after the ATG of *RBP35* coding sequence (CDS). No homologues were found for the two potential proteins encoded by uORFa and uORFb using a BLASTP search against the non-redundant database at the National Center for Biotechnology Information. In yeast, the transcriptional activator Gcn4p regulates the response to nutrient-poor conditions (46). *GCN4* mRNA contains four uORFs of only two or three codons in length. These uORFs inhibit the translation of downstream *GCN4* mRNA depending on the amino acid availability (46). Despite the differences in length compared to Gcn4p uORFs, Rbp35 uORFa and uORFb could play a regulatory role controlling Rbp35 protein levels.

To address this, we started by analysing their expression. We detected two transcripts within the *RBP35* 5′ UTR by northern blotting, in addition to the expected full-length mRNA of ∼2.5 kb (Figure 5C). These two transcripts of ∼1000 and ∼750 nt in length were named as uORF1 and uORF2, respectively. Both uORF1 and uORF2 were found to be polyadenylated (Figure 5D), and uORF1 was particularly induced under carbon starvation in *M. oryzae* WT strain. To confirm this, we analysed two different mutant strains with similar phenotypes that lack Rbp35, the original T-DNA mutant M35 and the knockout strain Δ*rbp35* (32). M35 contains a tandem T-DNA insertion, one within the *RBP35* CDS and the other within the 5′ UTR (Figure 5B, upper panel). By contrast, the *RBP35* locus in Δ*rbp35* possesses a 55 bp shorter 5′ UTR intron with an intact uORFa, and lacks part of uORFb and *RBP35* CDS (Figure 5B, bottom panel). Northern blots using an antisense strand probe of the *RBP35* 5′ UTR revealed that Δ*rbp35* was still able to generate uORF1, whereas M35 was unable to produce any of the *RBP35* transcripts (Figure 5E). To further confirm the production of these two uORFs, we cloned by reverse transcriptase-PCR (RT-PCR) and sequenced their 3′ ends using poly(A)+ RNA extracted from mycelia grown on MM-C (Supplementary Figure S5).

We observed that nutrient deprivation induces the degradation (or translation inhibition) of Rbp35A and the induction of two sense polyadenylated transcripts in the 5′ UTR *RBP35*. The lack of Rbp35A could influence the subunit composition of fungal CFI complex, and consequently, it would affect the 3′-end processing of target pre-mRNAs. The maintenance of Rbp35B suggested that either *M. oryzae* CFI complex is only formed by Rbp35B and CfI25, or Rbp35B plays an additional role under these nutritional conditions. M35 was unable to produce any of the two uORFs or *RBP35* mRNA since the first T-DNA insertion affected simultaneously the transcription of the three transcripts at the *RBP35* locus. These results and the presence of uORF1 in the *Δrbp35* mutant suggested that the ∼1000 nt uORF1 and uORF2 correlated with uORFa and uORFb, respectively.

### uORF1 and uORF2 are not required for Rbp35 proteolytic processing and turnover

To test whether uORF1 and uORF2 have a functional role in *M. oryzae*, *RBP35:mRFP* fusion constructs driven by its own promoter or *RP27* promoter were introduced into M35 (Figure 5F). The presence of Rbp35:mRFP was analysed by western blotting from cells grown on CM or carbon-depleted media (MM-C), where the two uORFs are expressed more abundantly. To our surprise, despite lacking M35 the full-length *RBP35* transcript (Figure 5E), a strongly induced protein of the expected size of Rbp35A:mRFP (∼73 kDa) was recognized by the anti-Rbp35 antibody in this mutant background in carbon-starved cells (Figure 5F). We confirmed by RT-PCR that this was due to a spontaneous translational fusion derived from the second T-DNA insertion (Supplementary Figure S4B). Using a second antibody generated against a peptide designed from the N-terminus of Rbp35 (Pep2, Supplementary Figures S2A and S4A), we confirmed that this translational fusion is not in frame with the amino end of Rbp35 (Figure 5G). After the splicing of the first intron, the mRNA fusion becomes in frame with *RBP35* CDS. This is probably the reason why the resulting recombinant protein that lacks a functional RRM domain is still recognized on MM-C by the anti-Rbp35 antibody generated against the entire protein. This fusion protein is not functional since M35 and Δ*rbp35* have a similar phenotype *in vitro* and in planta (32).

We observed that independently of the promoter and terminator constructs driving Rbp35 expression, the full-length Rbp35A:mRFP variant is always processed in M35 on CM (Figure 5F). In addition, the presence or absence of uORF1 and uORF2 is not interfering with the lack of Rbp35A in nutrient-poor conditions (Figure 5G). Consequently, we concluded that none of the two uORFs are regulating the proteolytic processing or turnover of Rbp35A in carbon-depleted cells.

### Rbp35 uORF1 regulates TOR-dependent fungal growth in *M. oryzae*

The TOR is a conserved signaling pathway in eukaryotes that acts as a critical sensor of the nutritional and energy status of the cell, and plays a key role as regulator of growth in response to environment by modulating primarily protein synthesis, autophagy and metabolism (47,48). In fact, *Δrbp35* showed accelerated growth rate compared to *M. oryzae* WT strain in the presence of ammonium tartrate as unique nitrogen source in MM, indicative of alterations in nitrogen metabolism (32). A transcriptome analysis of Δ*rbp35* revealed that several genes involved in the TOR pathway were down-regulated, correlating with the accelerated autophagy and higher tolerance to rapamycin observed in the Δ*rbp35* mutant (32).

**Figure 6.**
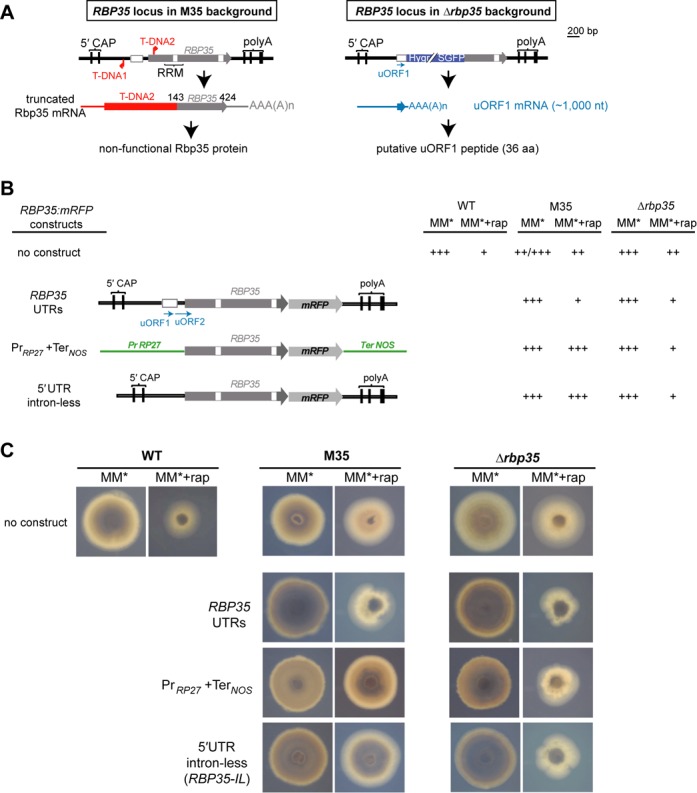
Rbp35-dependent fungal growth is regulated by uORF1 in the presence of rapamycin. (**A**) Description of *RBP35* locus in different mutant backgrounds. In M35, a truncated mRNA gives rise to a non-functional protein. In *Δrbp35*, only uORF1 mRNA is present. (**B**) *RBP35:mRFP* constructs in M35 and *Δrbp35* backgrounds exhibit different behaviour in rapamycin-containing medium. The *RBP35:mRFP* without the 5′ UTR intron or with a strong promoter (Pr*_RP27_*:*RBP35:mRFP*:Ter*_NOS_*) cannot reverse the phenotype of M35 to WT growth. Conversely, the growth reduction of *Δrbp35* strains containing these two constructs indicates a trans-acting role for uORF1. Scoring of growth rate:+++ for full growth, ++ for intermediate growth and + for minimal growth**.** (**C**) Images of WT strain, and M35 and *Δrbp35* mutants complemented with different *RBP35:mRFP* constructs in the presence of rapamycin. Pictures were taken at 5 dpi. MM*, minimal media containing 10 mM of ammonium tartrate as unique nitrogen source.

Here, we observed that uORF1 was highly induced in carbon-poor media (Figure 5C and D). To further analyse if uORF1 has a functional role in *M. oryzae*, we performed growth tests in the presence of rapamycin, a specific inhibitor of the TOR kinase, using M35 and Δ*rbp35* backgrounds complemented with different Rbp35:mRFP constructs (Figure 6A and B). As previously shown, M35 lacks both uORFs and part of *RBP35* CDS, while Δ*rbp35* still produces uORF1 but not uORF2 or *RBP35* CDS (Figures 5E and 6A).

In the absence of rapamycin, we confirmed that all strains showed a similar growth phenotype and grew like the WT strain in minimal media (32) (Figure 6B and C). Strikingly, in the presence of rapamycin, M35 strains complemented with Pr*_RP27_*:*RBP35:mRFP*:Ter*_NOS_*, which lack the 5′ UTR intron in the construct, showed an accelerated proliferation. This strain was not restoring the WT behaviour in the presence of rapamycin, suggesting a functional role for uORF1 and/or uORF2 under these conditions. The Δ*rbp35* mutant complemented with Pr*_RP27_*:*RBP35:mRFP*:Ter*_NOS_* restored the decreased growth rate shown by WT in rapamycin-containing medium, which highlighted the ability of uORF1 to play a role in trans.

To verify that the lack of the 5′ UTR intron was responsible for higher growth rates in the presence of rapamycin, a 5′ UTR intron-less construct C-terminally tagged with mRFP was generated (*RBP35-IL*). This construct, which maintained the rest of *RBP35* locus intact except for the 5′ UTR that lacked the 211 bp intron, was then introduced into M35 and Δ*rbp35* mutant strains (Figure 6B and C). The fast proliferation shown by M35/*RBP35-IL* strain indicated that the uORF1 and/or uORF2 regulated fungal growth in the presence of rapamycin. Likewise, the Δ*rbp35*/*RBP35-IL* strain showed a similar phenotype to the Δ*rbp35*/Pr*_RP27_*:*RBP35:mRFP*:Ter*_NOS_* strain, confirming the ability of uORF1 to play a role as a trans-acting factor.

The ability of Pr*_RP27_*:*RBP35:mRFP*:Ter*_NOS_* and *RBP35-IL* constructs in Δ*rbp35* background to reestablish WT growth in the presence of rapamycin confirms that Rbp35 uORF1 is sufficient to regulate TOR-dependent growth. It also points out that Rbp35 uORF1 can act as a trans-acting factor.

### The CFI proteins Hrp1 and CfI25 regulate fungal development and plant infection in *M. oryzae*

**Figure 7.**
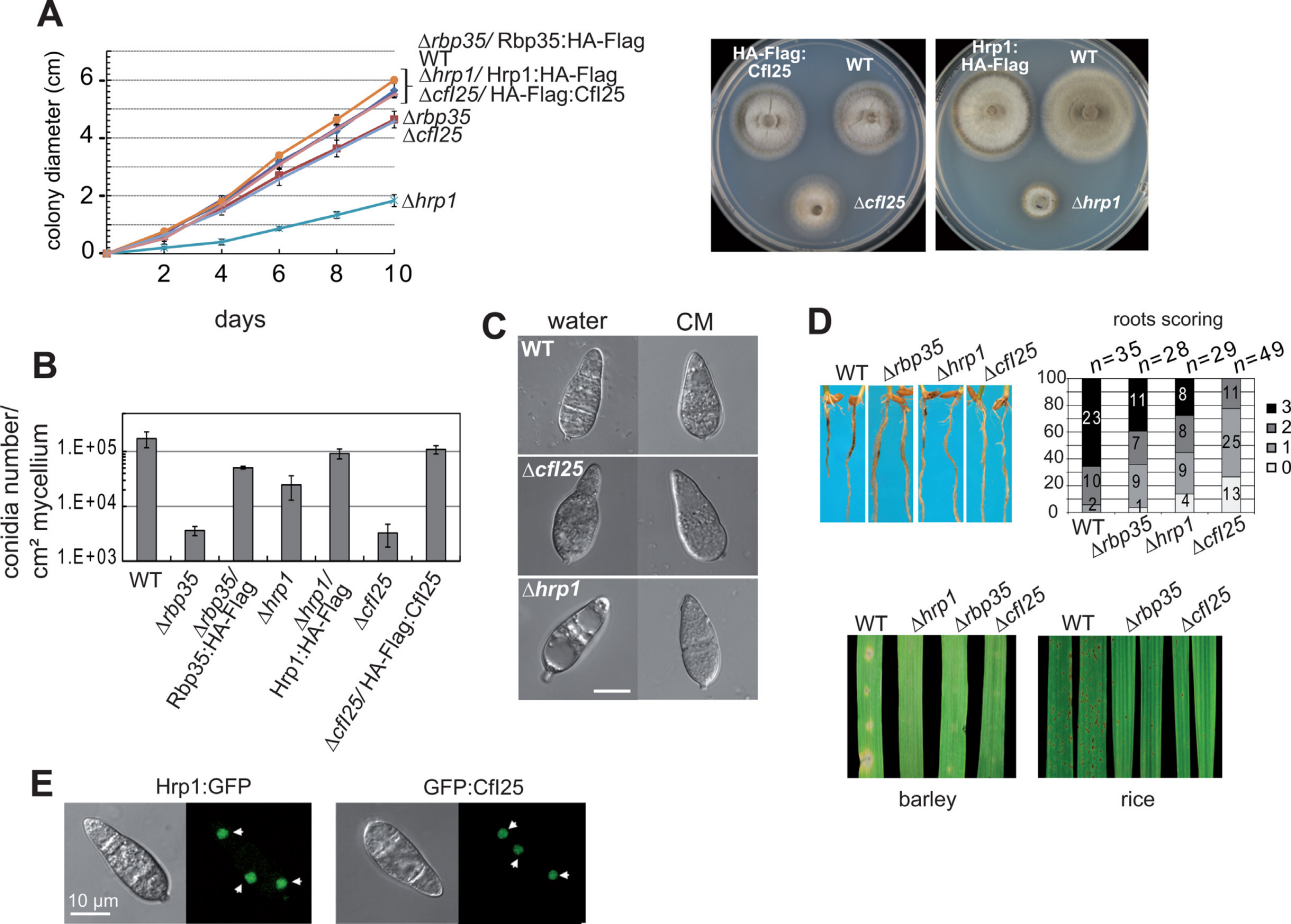
Hrp1 and CfI25 are involved in *M. oryzae* infection ability. (**A**) Growth rates (left panel, mean ± SD of three biological replica) and colony morphology (right panel) on CM. *Δhrp1* is severely affected in growth, while *ΔcfI25* growth rate is very similar to *Δrbp35*. (**B**) CfI25 is required for conidiation. Conidia of *Δhrp1* are degraded rapidly in the presence of water, probably due to the weakness of cell walls. Hrp1:HA-Flag and CfI25:HA-Flag rescue *Δhrp1* and *ΔcfI25* defects in growth and conidia number in water, respectively. (**C**) Conidiogenesis is more severely affected in *Δhrp1*. (**D**) *Δhrp1* and *ΔcfI25* are also defective in root (top panel) and leaf (bottom panel) infection. (**E**) Hrp1:GFP and CfI25:GFP show a steady-state nuclear localization. Nuclei are indicated by white arrowheads.

We were intrigued by the fact that filamentous fungi including *M. oryzae* possess Hrp1 and the Rbp35/CfI25 complex, proteins with apparently overlapping functions including alternative polyadenylation (24,32). Indeed, fission and budding yeast lack the Rbp35/CfI25 complex. Thus, we decided to generate the *HRP1* knockout strain to understand its involvement in *M. oryzae* biology (Figure 7 and Supplementary Figure S6A). Despite the growth and morphology defects showed by *M. oryzae Δhrp1*, this mutant was still viable in contrast to yeast (28) (Figure 7A). The *Δhrp1* strain exhibited a more severe phenotype than the *Δrbp35* mutant, suggesting that Hrp1 may play a more prominent role than Rbp35 in regulating the processing of pre-mRNAs. Conidia harvested in CM revealed that the *Δhrp1* mutant was not affected in conidia production since the observed number of conidia was within the range of the WT strain (∼10^5^ spores/cm^2^ of mycelium). However, we observed a reduction in the number of conidia produced by *Δhrp1* if they were harvested in water (Figure 7B), which suggested that conidial cytolysis occurred due to cell wall-associated defects (Figure 7C). Within a week, the *Δhrp1* mutant plates started to show mycelial lysis, normally associated with autolytic processes (data not shown). In accordance with the severe morphological defects, pathogenicity tests in barley leaves and rice roots showed that *Δhrp1* infection ability was significantly affected (Figure 7D). A functional C-terminal fusion Hrp1:GFP presented a steady-state nuclear localization under all the conditions tested (Figure 7E), which reflected a significant nuclear role for this protein.

**Figure 8.**
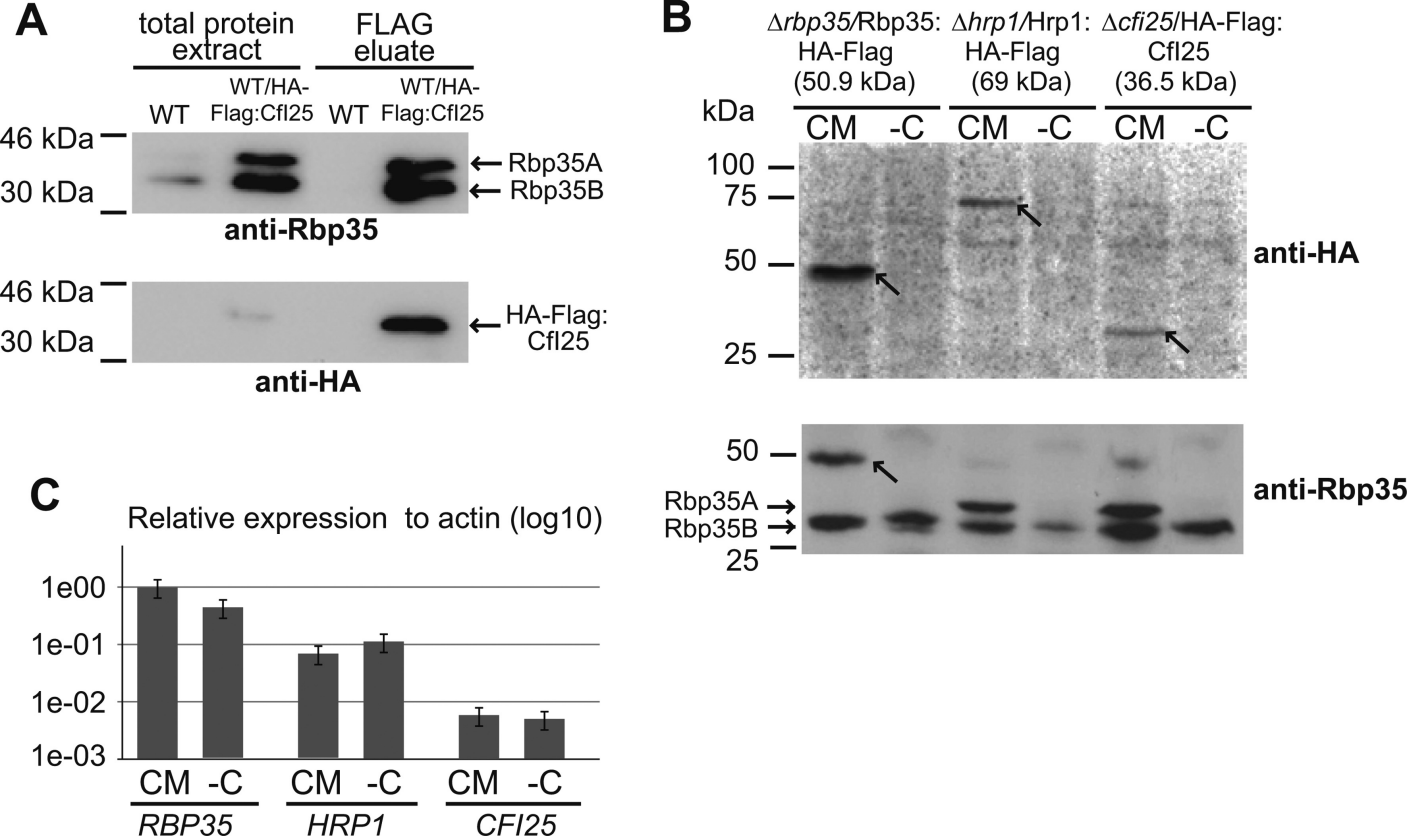
Characterization of CFI proteins in carbon-starved cells. (**A**) Co-immunoprecipitation experiments corroborate that CfI25 interacts *in vivo* with Rbp35A and Rbp35B. (**B**) Western blots of total protein extracts of *Δrbp35*, *Δhrp1* and *ΔcfI25* mutants complemented with HA-FLAG translational fusions of Rbp35, Hrp1 and CfI25, respectively. Remarkably, the three tagged proteins cannot be detected in carbon-depleted cells, only Rbp35B remains intact in the cell. Rbp35B is not visualized with the anti-HA antibody (Ab), but it is observed with anti-Rbp35 Ab (lower panel). (**C**) qPCR experiments with the same HA primers were carried out in the three complemented mutant strains. Graph shows that transcript levels of all HA-FLAG fusions remain unchanged in different nutritional conditions (mean ± SD of three biological replica). A direct correlation between transcript and protein levels is clearly observed for each of the proteins in CM, although in MM-C (-C) cannot be identified Rbp35A, Hrp1 or CfI25.

In addition, we investigated the fungal Rbp35/CfI25 complex. We deleted the *CFI25* gene in *M. oryzae* and confirmed that the phenotype shown by *ΔcfI25* was very similar to *Δrbp35* (32) (Figure 7A–D and Supplementary Figure S6B). The only difference between the two mutants was observed on roots, where *ΔcfI25* showed less necrotic symptoms compared to *Δrbp35* (Figure 7D). Similar to Rbp35 and Hrp1, the N-terminal GFP fusion of CfI25 exhibited a predominant nuclear localization (Figure 7E) (32). Significantly, co-immunoprecipitations using HA-Flag:CfI25 showed that CfI25 was able to interact *in vivo* with both isoforms of Rbp35 (Figure 8A).

To investigate if these two fungal CFI proteins are also regulated under nutritional stress, C-terminal HA-Flag translational fusions of Hrp1 and CfI25 were introduced in their respective mutant backgrounds and analysed by western blotting. These constructs were able to restore *Δhrp1* and *ΔcfI25* mutant defects (Figure 7A and B), which indicated that they are fully functional. Similarly to Rbp35, both Hrp1 and CfI25 could not be detected using anti-HA antibody in carbon-starved cells, suggesting that either nutrient-poor conditions tend to degrade CFI proteins in the fungal cell or their reduction is linked to general translation repression (Figure 8B). Despite these proteins were not detected by western blots, quantitative expression analysis revealed that their transcript levels remained constant in carbon-starved cells (Figure 8C).

Hrp1 and CFI25 play a significant role in *M. oryzae* biology although both proteins are non-essential for fungal viability. The stronger deficiencies observed in *ΔcfI25* during root infection suggests that the lack of CfI25 affects more severely transcripts associated with root infection compared to *Δrbp5*. It also indicates that CfI25 and Rbp35 are not playing an identical role in the cell. Furthermore, Rbp35, CfI25 and Hrp1 are subject to post-transcriptional regulation under nutritional stress, since their respective mRNA levels remain unchanged.

## DISCUSSION

The 3′-end processing of pre-mRNA is an essential step to form mature polyadenylated messengers. In humans and worms (*Caenorhabditis elegans*), more than half of the mRNAs present alternative polyadenylation signals (49), which manifests the relevance of this post-transcriptional regulatory mechanism, likely to affect mRNA stability, translation and translocation (17). CFI proteins play an important role in alternative polyadenylation. Here, we provide evidence that multiple layers of regulation control CFI proteins in filamentous fungi, and particularly, in the rice blast fungus *M. oryzae*.

A correlation exists between mammalian and *M. oryzae* CFI complexes in that both are composed of protein variants with different molecular weights. Mammalian CFI_m_ is an heterotetrameric protein complex composed of two molecules of CFI_m_25 and two other molecules of CFI_m_59, CFI_m_68 kDa or CFI_m_72 kDa (20,50,51). Two paralogue genes, CPSF7 and CPSF6, encode the 59 and 68 kDa CFIm subunits, respectively. To our knowledge, the origin of the CFI_m_72 kDa protein is unknown. In *M. oryzae*, CFI complex is formed by CfI25 and two Rbp35 isoforms that derived from a single gene locus, the full-length protein (Rbp35A) and its processed form (Rbp35B) (32).

The presence of two protein isoforms of Rbp35 suggests that several types of CFI complexes could process differentially pre-mRNA 3′ ends in *M. oryzae*. Interestingly, the processing of Rbp35 has been maintained within the ascomycota fungi, suggesting that this post-transcriptional regulatory event occurred before the diversification of this phylum. In order to investigate this and to identify Rbp35 processing site, several Rbp35:mRFP variants were constructed by mutating the six Arg of the RGG module and generating C-terminally truncated forms of the protein. Our results were conclusive; the processing of Rbp35 occurs after the RGG module. The three Rbp35:mRFP variants Rbp35-noRGG, Rbp35Δ_239–424_ and Rbp35Δ_318–424_ accumulate partially in the cytoplasm, which confirms that nuclear transport signals are present in the C-terminal end of Rbp35. Consistent with this, a bipartite NLS has been predicted in this protein (32) (Figure 1A and Supplementary Figure S2A). As described for other RGG-containing RNA-binding proteins (43), the enrichment of Rbp35-noRGG in the periphery of the nucleus suggests that Arg methylation of RGG tripeptides could regulate Rbp35 nucleocytoplasmic transport. Alternatively, the point mutation on the last RGG within the NLS-1 could be affecting its recognition by importins. Due to the location of the processing site, possibly the NLS-1 becomes more exposed to recognition by importins after the cleavage.

Functional analysis of Rbp35 auxiliary domains showed that the two Rbp35:mRFP constructs with C-terminal truncations recovered some of *Δrbp35* deficiencies and restored partially conidiation and conidiogenesis. They were unable to recover the production of pigmented metabolites, suggesting the relevance of the auxiliary RGG and Met-Asn-Gly domains in Rbp35 function. Structural studies have assigned a role in RNA recognition to the Arg of the RGG region in the RNA-binding protein FMRP (52), and can also participate in protein-protein interactions (53). Similarly, we assume that the RGG domain of Rbp35 can be playing a role in recognition of pre-mRNAs and interaction with other proteins, i.e. CfI25 or itself.

The high expression levels of Rbp35Δ_239–424_ and Rbp35Δ_318–424_ in the cell suggest the presence of regulatory motifs in the 3′ of the *RBP35* gene or the carboxy end of the protein. To follow-up this finding, we replaced *RBP35* native UTRs by the *RP27* strong promoter and *NOS* terminator in several *RBP35:mRFP* gene constructs. Subsequent qPCR analysis revealed that the mRNA levels did not correlate with their protein abundance, which indicates that the C-terminal end of Rbp35 acts as an internal sensor to control its own protein levels. In addition, CHX treatment identified degradation signals present in the RGG region, which suggests that Rbp35 cellular levels are the result of combined regulatory mechanisms balancing protein synthesis and degradation. Indeed, it is common to find strong autoregulatory circuits in RNA-binding proteins. They can regulate their own mRNA translation and turnover in addition to other mRNAs that encode additional RNA-binding proteins, thus becoming master regulators of RNA-binding protein networks (54). A translational inhibitory mechanism has been described for the yeast RNA-binding Scd6 required for translation and mRNA degradation (55). The interaction of the C-terminal RGG domain of Scd6 with eIF4G represses translation initiation, and other yeast RGG-containing proteins also share this inhibitory mechanism mediated by their RGG domain, including Npl3 and Sbp1 (55). In this context, Rbp35 C-terminal end is important to fulfil this regulatory mechanism.

A comparative proteomic approach using WT and mutant strains gave us a deeper insight into how the lack of Rbp35 was affecting cellular proteins. We classified the 27 proteins up-/down-regulated in *Δrbp35* into functional groups to better understand its phenotypic defects. Several enzymes required for melanin and flavonoid synthesis are down-regulated in *Δrbp35*. Melanin is required for appressorium function and it also protects fungal hyphae from oxidative stress (56). The deficiencies found in melanin production correlate with the defects in pigmentation of Δ*rbp35*. In *M. oryzae*, mutants unable to produce melanin are non-pathogenic (57). This could partially explain the pathogenicity deficiencies displayed by the *Δrbp35* mutant in leaf infection assays. Very little is known about the involvement of flavonoids in *M. oryzae* infection process. Remarkably, three down-regulated proteins in *Δrbp35* show no changes or a 2-fold increase in mRNA levels, which suggests a direct or indirect role for Rbp35 in their translation or degradation rates. One of those three proteins (MGG_03506) contained a predicted RRM, which is in accord with above-mentioned model of Rbp35 as a regulator of other RNA-binding proteins (54).

We observed the disappearance of Rbp35A in carbon-starved cells. In metazoans, the heterotetramer composition of CFI_m_ possibly influences a poly(A) site preference within the same pre-mRNA (20). Similarly, the disappearance of the Rbp35 full-length isoform may be influencing the stoichiometry of the fungal CFI complex, affecting the selection of proper poly(A) sites in pre-mRNA targets and altering their expression.

Remarkably, an additional post-transcriptional mechanism has been identified within *RBP35* 5′ leader region. Two sense polyadenylated transcripts (uORF1 and uORF2) are detected by northern blots in *RBP35* 5′UTR. uORF1 is particularly induced in carbon-starved cells. A bioinformatic analysis also predicts two uORFs (uORFa and uORFb) within the *RBP35* 5′ UTR intron. In the *Δrbp35* mutant, the deleted region includes the predicted uORFb and part of *RBP35* CDS, but not uORFa. The uORFa DNA sequences remain intact in this mutant. Northern blots correlate the ∼1000 nt uORF1 with the predicted uORFa. Our current working model is that uORF1 and uORF2 (∼750 nt) are transcribed from different start sites within *RBP35* promoter, which would explain their differences in size. Further studies will be needed to identify the transcription start sites, and if uORF2 plays any role in the cell.

The T-DNA insertion line M35 is impaired in the formation of uORF1 and uORF2, and the full-length *RBP35* mRNA. We have used this mutant and *Δrbp35* backgrounds to dissect in more detail the potential role of uORF1. According to the accelerated growth shown by M35 and normal behaviour of *Δrbp35* in the presence of rapamycin complemented either with the intron-less construct Rbp35-IL or with Pr*_RP27_*:*RBP35:mRFP*:Ter*_NOS_*, the uORF1 can influence Rbp35-mediated regulation of the TOR pathway. In fact, it can also act as a trans-acting factor based on the WT phenotype shown by *Δrbp35* strains harbouring these same constructs. Therefore, it is clear that the uORF1 is functional in *M. oryzae*, and that regulates the TOR cascade by regulating Rbp35.

**Figure 9.**
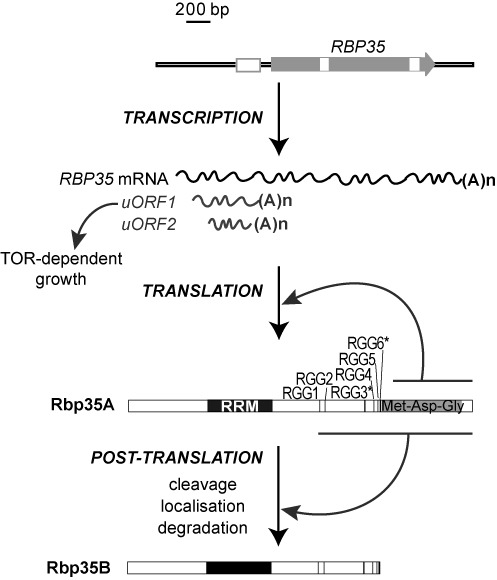
Multi-layer regulation of Rbp35 in *M. oryzae*. Three different transcripts are produced at the *RBP35* gene locus, the *RBP35* mRNA and two smaller RNAs derived from the 5′ UTR, namely, *uORF1* and *uORF2*. The *uORF1* controls *M. oryzae* growth activated by the TOR pathway. Translation of the mRNA generates the full-length isoform of the protein, Rbp35A. This step is regulated by its C-terminal Met-Asn-Gly domain. Post-translational processing of Rbp35A generates a second isoform Rbp35B, which interacts with both Rbp35A and CfI25 within the fungal CfI25/Rbp35 complex. The processing site of the protein is after the RGG region. Cleavage efficiency, localization and degradation processes are regulated by the RGG domain.

uORFs of several fungal genes have been characterized in detail (46,58). The occurrence of uORFs normally affects downstream initiation efficiency of the main protein CDS, and consequently the translation rate of the respective protein, although it can also affect mRNA stability by promoting NMD (58,59). The uORF1 can be inhibiting or promoting translation reinitiation of *RBP35* mRNA, and contributing to the maintenance of Rbp35 protein levels in the fungal cell (Figure 9). This regulation is possibly modulated by the TOR signaling cascade, which can promote translation reinitiation of uORF-containing mRNAs via phosphorylation of eIF3h (60).

*Saccharomyces cerevisiae* Hrp1 is required for the selection of a proper poly(A) site by positioning itself upstream of the cleavage site (24), and regulates alternative polyadenylation. It is a nucleocytoplasmic shuttling protein, and arginine methylation is required for its efficient nuclear export (61). In *M. oryzae*, Hrp1 shows a strong nuclear localization although a faint fluorescence background can be detected in the *Δhrp1*/Hrp1:GFP strain, which suggests that *M. oryzae* Hrp1 could also be localized in the cytoplasm. This correlates with the localization pattern of *F. graminearum* Hrp1, where it can be observed in both compartments (62). Similar to Hrp1, *M. oryzae* CfI25 also plays a significant role in *M. oryzae* development and pathogenesis. We have confirmed that CfI25 interacts with the two Rbp35 isoforms, corroborating the presence of Rbp35A, Rbp35B and CfI25 in this complex. *Magnaporthe oryzae* CfI25 shows a steady-state nuclear localization. In metazoans, CFI_m_25 and CFI_m_68 are also nuclear at steady states, although both are nucleocytoplasmic shuttling proteins (63). Based on the differences found in the ability to infect rice roots by *ΔcfI25* and Δrbp35, *M. oryzae* CfI25 and Rbp35 may play different roles within the Rbp35/CfI25 complex or have additional roles.

A tight regulation of alternative polyadenylation is essential for cellular development. Here, we demonstrate that cellular concentration of CFI mRNAs is a limited indicator of their protein abundance. Different post-transcriptional levels regulate Rbp35/CfI25 complex and Hrp1 in *M. oryzae*, some of which are also conserved in several ascomycetous fungi. With respect to Rbp35, these include C-terminal processing, RGG-dependent localization, C-terminal autoregulatory domain and regulation by uORF1 of Rbp35-dependent TOR kinase pathway (Figure 9). The drastic reduction of fungal CFI proteins Hrp1, CfI25 and Rbp35A in carbon-starved cells suggests that alternative polyadenylation is altered, affecting consequently the mRNA networks controlled by these proteins.

Our findings uncover broad and multi-layer regulatory mechanisms controlling fungal-specific polyadenylation factors, which have profound implications in pre-mRNA maturation. These findings increase our understanding of how CFI proteins, and consequently, alternative polyadenylation, are tuned in the fungal kingdom. Future work will focus on the identification of mRNA networks regulated by different CFI proteins in *M. oryzae*, and their involvement in plant pathogenesis. Our approach to understanding the regulation and function of CFI proteins in *M. oryzae* provides a rich avenue for studying the use fungal-specific components of the polyadenylation machinery as novel targets to control fungal diseases.

## SUPPLEMENTARY DATA

Supplementary Data are available at NAR Online.

SUPPLEMENTARY DATA
