# Supplementary material for: Multilayer regulatory mechanisms control cleavage factor I proteins in filamentous fungi
Source: Nucleic Acids Res. 2014 Dec 16;43(1):179–95. doi: 10.1093/nar/gku1297 (PMC4288187; doi:10.1093/nar/gku1297)
Supplement: SUPPLEMENTARY DATA [file supp_43_1_179__index.html]

Multilayer regulatory mechanisms control cleavage factor I proteins in filamentous fungi — SUPPLEMENTARY DATA 

# Multilayer regulatory mechanisms control cleavage factor I proteins in filamentous fungi

## SUPPLEMENTARY DATA

**Files in this Data Supplement:**

- SUPPLEMENTARY DATA
