# Supplementary material for: Multilayer regulatory mechanisms control cleavage factor I proteins in filamentous fungi
Source: Nucleic Acids Res. 2014 Dec 16;43(1):179–95. doi: 10.1093/nar/gku1297 (PMC4288187; doi:10.1093/nar/gku1297)
Supplement: SUPPLEMENTARY DATA [file supp_gku1297_nar-01971-a-2014-File011.pdf]

Figure S1

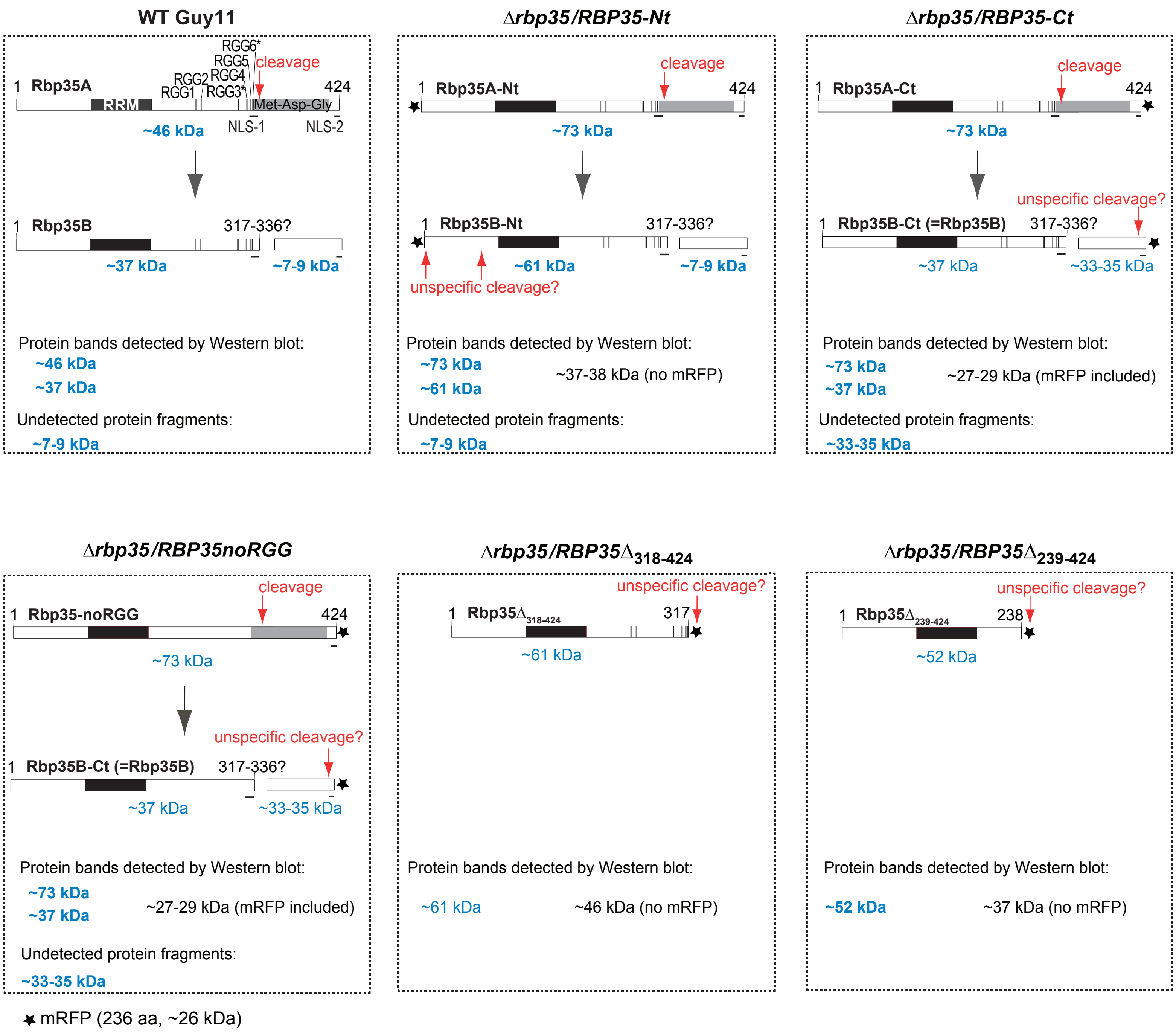

Figure S1. Schematic illustration of Rbp35 processing. Representation of predicted and identified protein band sizes of Rbp35 and Rbp35:mRFP variants by Western blotting (Figure 1B). Numbers in blue represent predicted protein band sizes. Based on the immunoblots, all Rbp35:mRFP variants have an additional unspecific cleavage, whose position varies depending on the protein construct.

Figure S2

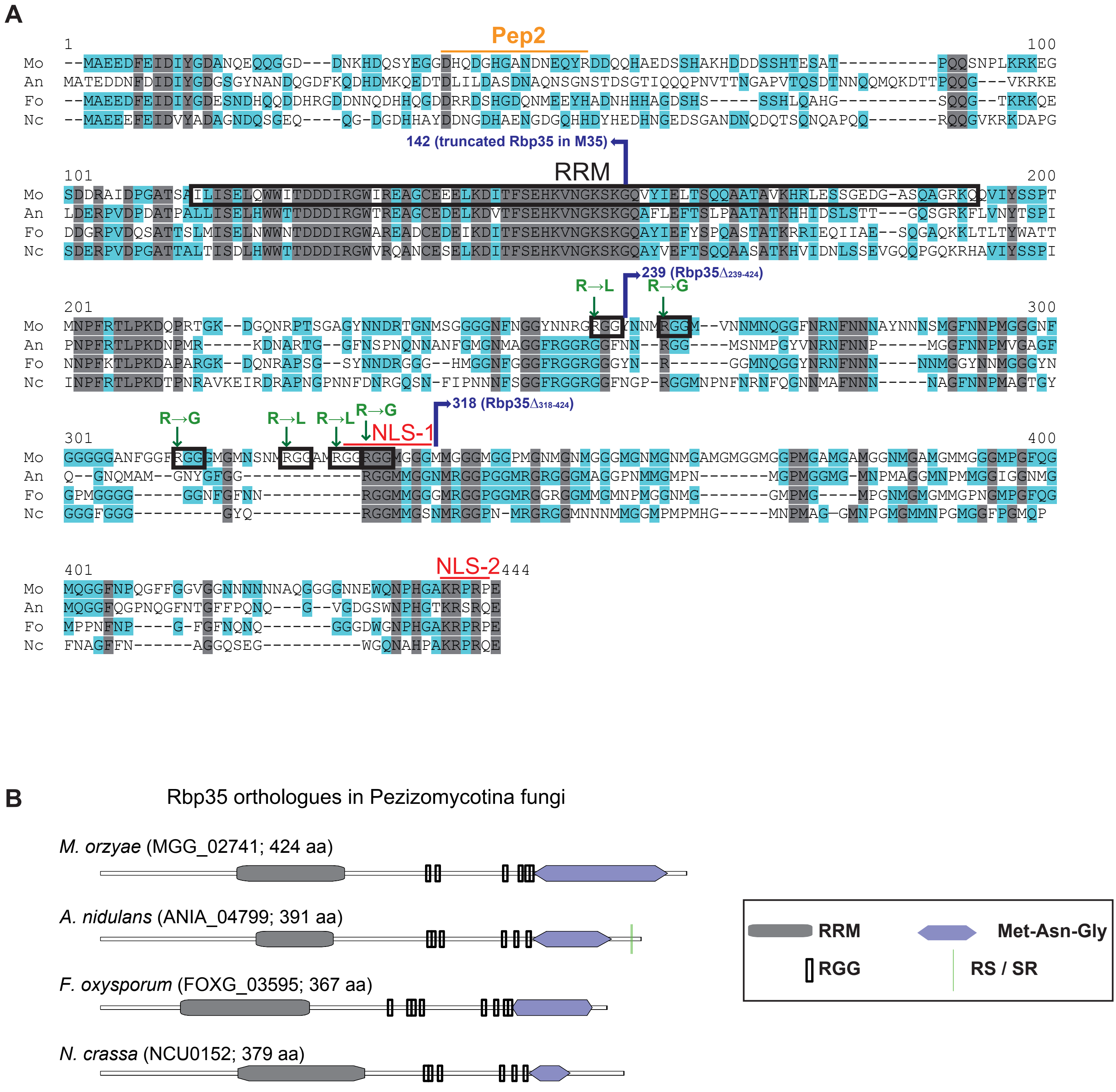

Figure S2. Rbp35 orthologues in Pezizomycotina fungi. (A) Sequence alignment of Rbp35 proteins from *M. oryzae* Guy11 (Mo), *A. nidulans* FGSC A4 (An), *F. oxysporum* f.sp. *lycopersici* 4287 (Fo) and *N. crassa* FGSC 2489 (Nc). The numbers represents the aa position of the consensus alignment. Conservative amino acid substitutions are indicated in colored boxes (blue, similar; grey, identical). The RRM and RGG motifs of *M. oryzae* Rbp35 are outlined with a black box. In *M. oryzae* Rbp35, aminoacid substitutions of Rbp35-noRGG are indicated in green and deleted regions of Rbp35 truncated variants are indicated in purple. Bi-partite nuclear localisation signal (NLS) is highlighted in red. The second Rbp35 antibody (anti-Pep2) was raised against the Pep2 sequence. **(B)** Protein domain structure of Rbp35 orthologues.

Figure S3

A

	20 min extraction at 80° C			3 h extraction at 80° C		
	Absorbance (λ) ± SD			Absorbance (λ) ± SD		
	**470 nm**	**500 nm**	**650 nm**	**470 nm**	**500 nm**	**650 nm**
Guy 11	0.66±0.05	0.51±0.04	0.18±0.02	0.90±0.08	0.71±0.09	0.29±0.09
Δ *rbp35*	0.42±0.01	0.33±0.01	0.10±0.01	0.54±0.02	0.42±0.03	0.14±0.02
	mean percentage			mean percentage		
Guy 11	100	100	100	100	100	100
Δ *rbp35*	64.06	64.59	59.66	59.82	59.53	48.61

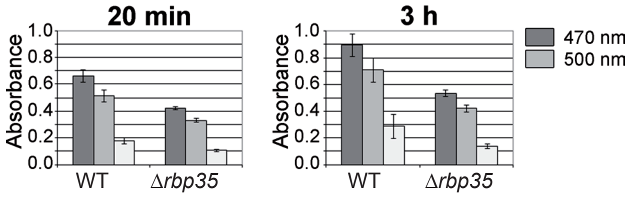

B

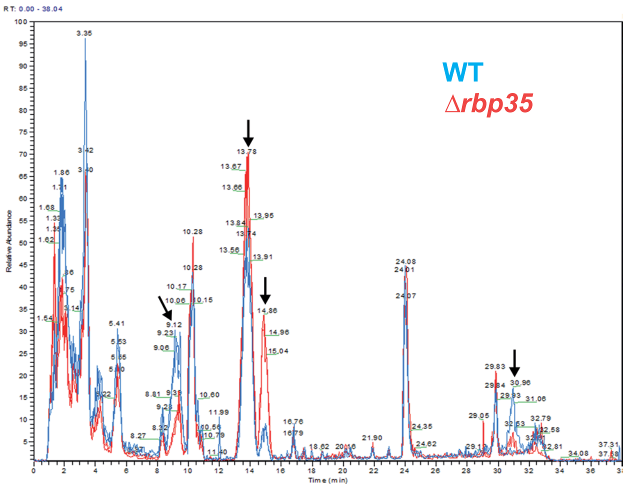

Figure S3. Melanin content and flavonoids profiles in WT and *Δrbp35* strains. (A) Clear reduction in melanins (~36-52%) is detected by colorimetric assays in *Δrbp35*. **(B)** A HPLC profile of flavonoid extracts from wild type (WT) Guy11 strain and the *Δrbp35* mutant shows the appearance and disappearance of peaks (arrows) in *Δrbp35*, corroborating alterations in the synthesis of these type of compounds.

Figure S4

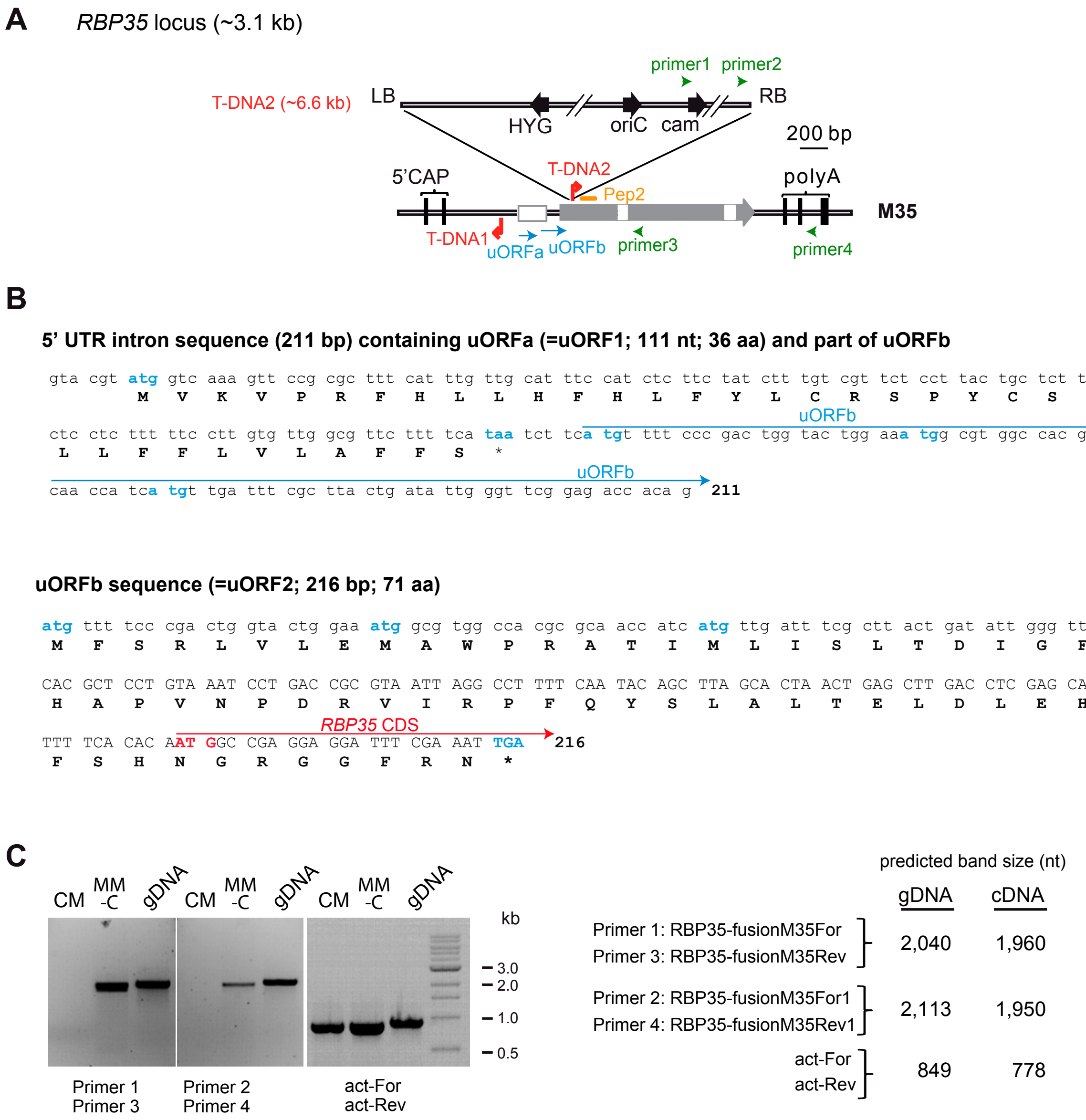

Figure S4. Description of Rbp35 uORFs and the observed Rbp35 protein band in M35. (A) Diagram depicting the *RBP35* locus in M35. **(B)** DNA and protein sequences of the two predicted uORFs. The 211 bp 5' UTR intron contains the entire uORFa and partially uORFb. The uORFb ovelaps 26 bp with the coding sequence (CDS) of *RBP35*. Exonic DNA sequences are represented in upper case, while intronic sequences in lower case. Northern experiments using M35 and *Δrbp35* mutants correlate uORFa and uORFb with the transcripts found in the *RBP35* 5'UTR uORF1 and uORF2, respectively (Figure 5E). **(C)** Confirmation by RT-PCR of the generation of a fusion transcript between the second T-DNA insertion and the *RBP35* CDS. This fusion transcript is not detected by Northern blotting using the *RBP35* 5'UTR as a probe (Figure 5E). The transcript generated in this M35 locus is not in frame with *RBP35* CDS since the generated fusion protein is not recognized by an antibody raised specifically against the amino end of Rbp35. After the splicing of the first intron of *RBP35*, this translational fusion restores his frame with the *RBP35* CDS. The second Rbp35 antibody was raised against the sequence DHQDGHGANDNEQYR (Pep2; Figure S1A).

Figure S5

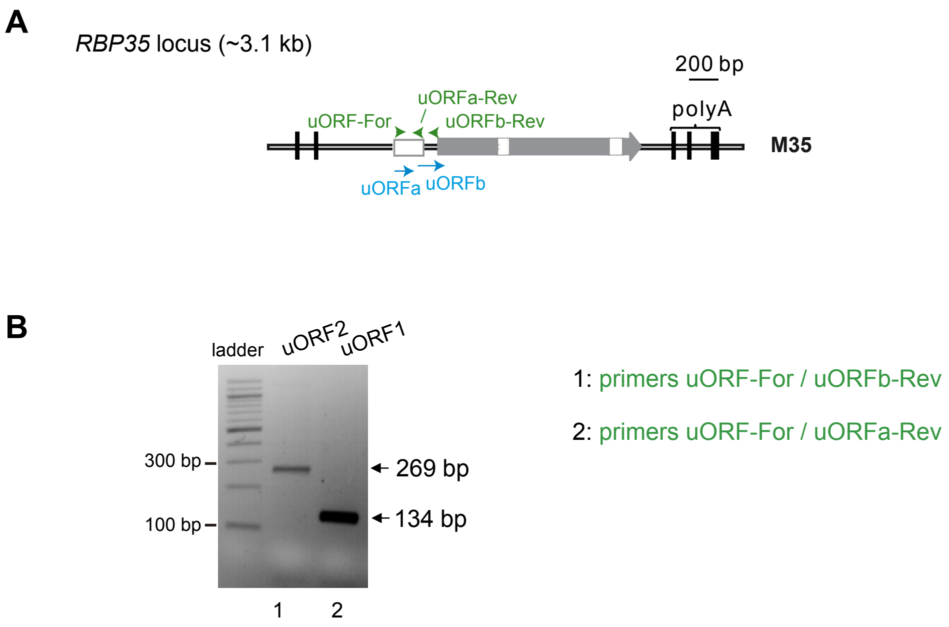

Figure S5. Isolation of *uORF1* and *uORF2* 3’ ends by RT-PCR. (A) Diagram depicting the *RBP35* locus in *M. oryzae*. **(B)** The *uORF1* and *uORF2* transcripts detected by Nothern blotting in *RBP35* 5’UTR were confirmed by RT-PCR using poly(A)+ RNA and primers that specifically amplified the 3’ ends of *uORF1* and *uORF2* transcripts and not *RBP35* mRNA. PCR products were confirmed by sequencing.

Figure S6

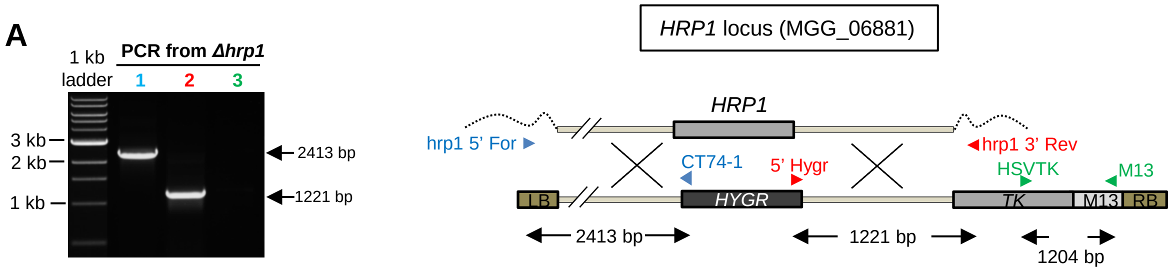

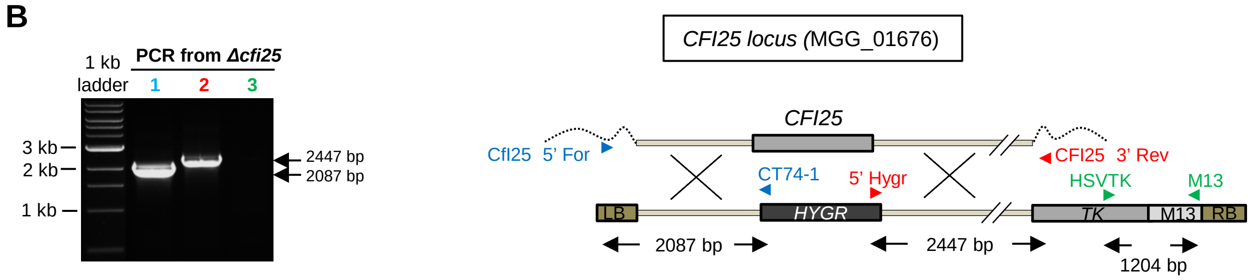

Figure S6. Replacement of *M. oryzae HRP1* and *CFI25* genes. Schematic diagrams showing targeted gene deletion strategies of *HRP1* (A, right panel) and *CFI25* (B, righ panel). Single PCR products of expected band sizes confirmed *HRP1* and *CFI25* gene replacements using genomic DNA from *Δhrp1* and *ΔcfI25* strains and the following pair of primers:

- for *Δhrp1*: hrp1 5' For/CT74-1 (lane 1), hrp1 3' Rev/5' Hygr (lane 2), and HSVTK/M13 (lane 3).
- for *ΔcfI25:* CFI25 5' For/CT74-1 (lane 1), CFI25 3' Rev/5' Hygr (lane 2), and HSVTK/M13 (lane 3).

Absence of a PCR product with HSVTK and M13 primers indicated the lack of an ectopic insertion due to the presence of an inactive timidine kinase (TK) gene.

Table S1. List of differentially expressed proteins in *Δrbp35* identified by MS after separation in two dimensional gels.

	Spot n.	Protein	Description/orthologues	MS[1]	FC-p[2]	FC-m[3]	References
	**Primary and secondary metabolism (18 proteins)**						
**DOWN**	1	MGG_12981	cupin domain/**BinA** (e-22; *A. nidulans*)	98	-6.9±0.2	-2.4 / -2.4	
	2	MGG_02254	SAM-dependent methyl transferase/**Crg1** (e-6; yeast)	69	-5.8±0.1	-2.3 / -2.5	
	3	MGG_08201*	Glutathione-dependent formaldehyde-activating enzyme; **Mss4**-like (yeast)	56	-3.5±0.3	1.0 / -1.4	
	4	MGG_06917	GTP cyclohydrolase/synthesis flavoproteins/**Rib1** (e-5; yeast)	90	-3.0±0.0	-1.2 / -1.3	
	5	MGG_13188	NADPH-oxidoreductase / K+channel subunit 2	124	-2.9±0.1	no data	
	6	MGG_01084	glycerol dehydrogenase/**Tdh1** (e-116; yeast)	237	-2.8±0.5	-1.3 / -1.4	
	7	MGG_12868	enoyl-coA hydratase/**EchA** (e-101; *A. nidulans*)	92	-2.7±0.1	no data	(1)
	8	MGG_10583*	12-oxophytodienoato reductase 1/flavonoid Mb/**Oye2** (e-55; yeast)	175	-2.7±0.1	2.1 / 2.2	
	9	MGG_09297	alcohol dehydrogenase/**TdiC** (e-45; *A. nidulans*)	57	-2.6±0.2	-2.0 / -2.2	(2)
	10	MGG_02252*	melanin synthesis/ **3THNR** (*M. oryzae*)	272	-2.3±0.2	-1.5 / -1.7	(3)
	11	MGG_10252	RmlC-like cupin domain/**oxdC** (yeast)	88	-2.2±0.1	-1.1 /-1.3	(4)
	12/12b	MGG_05059*	scytalone dehydratase/ **SDH** (*M. oryzae*)	85	-2.1±0.4	-1.3 / -1.5	(3)
	13	MGG_05283*	uricase / **UaZ** (e-113; *A. nidulans*)	122	-2.1±0.1	-1.1 / -1.4	(5,6)
	14	**MGG_02921***	glycerol dehydrogenase/ **GCY1** (e-61; yeast)	136	-2.1±0.2	2.0 / 2.0	
**UP**	15	MGG_10088*	ADP-ribose binding module/ **Poa1** (e-7; yeast)	109	6.5±0.1	2.0 / 2.3	(7)
	16	MGG_08440	trichothecene acetyl-transferase/**Ayt1** (e-51; yeast)	96	3.4±0.1	1.5 / 1.6	(8)
	17	MGG_15774	Val-leu-Ile-synthesis/**ILV5** (e-156; yeast)	124	3.3±0.2	1.5 / 1.5	
	18	MGG_08359*	Oxoglutarate/iron-dependent dioxygenase/flavonol synthase	305	1.9±0.2	2.8 / 3.1	
	**Others (9 proteins)**						
**DOWN**	19	**MGG_09757***	glucosyl hydrolase GH31/**Rot2** (e-21; yeast)	91	-12.3±0.0	1.5 / 1.6	
	20	MGG_09075*	BolA superfamily / **Aim1** (e-16; yeast)	57	-6.5±0.0	1.0 / -1.0	
	21	MGG_08622	nucleoside diphosphate kinase/**SwoH** (e-63; *A. nidulans*)	91	-3.0±0.5	no data	(9)
	22	**MGG_03506***	RRM protein/ P-granules formation/ **Pub1** (e-6; yeast)	104	-2.5±0.1	1.2 / 1.2	
	23	MGG_17062*	thioredoxin-like fold	75	-2.4±0.2	no data	
	24	MGG_04337*	peroxidase-catalase/**MgKATG1=CPXA** (*M. oryzae*)	161	-2.0±0.0	1.0 / 1.2	(10,11)
**UP**	25	MGG_15048	NAD-dependent deacetylase sirtuin-5/ **Hst2** (e-13; yeast)	76	2.4±0.1	no data	(12)
	26	MGG_07752	mitochondrial F1 ATPase subunit alpha / **ATP1** (e-241/ yeast)	106	4.3±0.2	1.2 / 1.3	(13)
	27	MGG_07066*	MCP2 mitochondrial carrier/ **AGC1** (e-106; yeast)	62	3.1±0.0	3.2 / 3.3	

*: expression of these genes has been analysed by qPCR (Figure 4B). Expression of genes in bold is up-regulated or remains unchanged in the *Δrbp35* mutant.

[1]Mascot scores (MS) higher than 56 are significant ($p<0.05$);

[2]Protein fold change obtained in the proteomic experiment (FC-p)

[3]mRNA fold change of two oligoarrays from microarray experiment (FC-m) (14)

REFERENCES (Table S1)

1. Maggio-Hall, L.A. and Keller, N.P. (2004) Mitochondrial beta-oxidation in Aspergillus nidulans. *Molecular Microbiology*, **54**, 1173-1185.
2. Hoffmeister, D., Bouhired, S., Weber, M., Kempf-Sontag, A. and Keller, N.P. (2007) Accurate prediction of the Aspergillus nidulans terrequinone gene cluster boundaries using the transcriptional regulator LaeA. *Fungal Genetics and Biology*, **44**, 1134-1145.
3. Chumley, F.G. and Valent, B. (1990) Genetic analysis of melanin deficient, nonpathogenic mutants of *Magnaporthe grisea*. *Molecular Plant-Microbe Interactions*, **3**, 135-143.
4. Nielsen, J., David, H., Ozcelik, I.S. and Hofmann, G. (2008) Analysis of *Aspergillus nidulans* metabolism at the genome-scale. *BMC Genomics*, **9**.
5. Oestreicher, N. and Scazzocchio, C. (1993) Sequence, Regulation, and Mutational Analysis of the Gene Encoding Urate Oxidase in *Aspergillus nidulans*. *Journal of Biological Chemistry*, **268**, 23382-23389.
6. Gournas, C., Oestreicher, N., Amillis, S., Diallinas, G. and Scazzocchio, C. (2011) Completing the purine utilisation pathway of Aspergillus nidulans. *Fungal Genetics and Biology*, **48**, 840-848.
7. Shull, N.P., Spinelli, S.L. and Phizicky, E.M. (2005) A highly specific phosphatase that acts on ADP-ribose 1 "-phosphate, a metabolite of tRNA splicing in *Saccharomyces cerevisiae*. *Nucleic Acids Research*, **33**, 650-660.
8. Satrustegui, J., Contreras, L., Gomez-Puertas, P., Iijima, M., Kobayashi, K. and Saheki, T. (2007) Ca2+ activation kinetics of the two aspartate-glutamate mitochondrial carriers, aralar and citrin - Role in the heart malate-aspartate NADH shuttle. *Journal of Biological Chemistry*, **282**, 7098-7106.
9. Lin, X.R., Momany, C. and Momany, M. (2003) SwoHp, a nucleoside diphosphate kinase, is essential in Aspergillus nidulans. *Eukaryotic Cell*, **2**, 1169-1177.
10. Skamnioti, P., Henderson, C., Zhang, Z., Robinson, Z. and Gurr, S.J. (2007) A Novel Role for Catalase B in the Maintenance of Fungal Cell-Wall Integrity During Host Invasion in the Rice Blast Fungus *Magnaporthe grisea*. *Molecular Plant-Microbe Interactions*, **20**, 568-580.
11. Zamocky, M., Furtmüller, P.G., Bellei, M., Battistuzzi, G., Stadlmann, J., Vlasits, J. and Obinger, C. (2009) Intracellular catalase/peroxidase from the phytopathogenic rice blast fungus *Magnaporthe grisea*: expression analysis and biochemical characterization of the recombinant protein. *Biochemistry Journal*, **418**, 443–451.
12. Brachmann, C.B., Sherman, J.M., Devine, S.E., Cameron, E.E., Pillus, L. and Boeke, J.D. (1995) The Sir2 Gene Family, Conserved from Bacteria to Humans, Functions in Silencing, Cell-Cycle Progression, and Chromosome Stability. *Genes & Development*, **9**, 2888-2902.
13. Gowda, M., Venu, R.C., Raghupathy, M.B., Nobuta, K., Li, H.M., Wing, R., Stahlberg, E., Couglan, S., Haudenschild, C.D., Dean, R. *et al.* (2006) Deep and comparative analysis of the mycelium and appressorium transcriptomes of *Magnaporthe grisea* using MPSS, RL-SAGE, and oligoarray methods. *BMC Genomics*, **7**.
14. Franceschetti, M., Bueno, E., Wilson, R.A., Tucker, S.L., Gómez-Mena, C., Calder, G. and Sesma, A. (2011) Fungal Virulence and Development Is Regulated by Alternative Pre-mRNA 3’ End Processing in *Magnaporthe oryzae*. *PLoS Pathogens*, **7**, e1002441.

Table S2. Strains used in this study.

**Strain name/ description**	**Parental strain**	**Resistance**	**Fluorescence markers/tags**	**Reference**
wild type rice isolate Guy11				(1)
Δrbp35	Guy11	hygr	GFP	(2)
***Δrbp35* (native 5' and 3' regions)**				
Δrbp35/ Rbp35-Nt (=RBP35-mRFP-N; native UTRs)	*Δrbp35*	hygr, sulph	GFP, N-terminal mRFP (cherry)	(2)
Δrbp35/ Rbp35-Ct (=RBP35-mRFP-C; native UTRs)	*Δrbp35*	hygr, sulph	GFP, C-terminal mRFP (cherry)	(2)
Δrbp35/ Rbp35-noRGG (native UTRs)	*Δrbp35*	hygr, sulph	GFP, C-terminal mRFP (cherry)	this study
Δrbp35/ $Rbp35_{\Delta 314\text{-}424}$ (native UTRs)	*Δrbp35*	hygr, sulph	GFP, C-terminal mRFP (cherry)	this study
Δrbp35/ $Rbp35_{\Delta 239\text{-}424}$ (native UTRs)	*Δrbp35*	hygr, sulph	GFP, C-terminal mRFP (cherry)	this study
Δrbp35/ Rbp35:HA-Flag(=RBP35-HA-Flag;native UTRs)	*Δrbp35*	hygr, sulph	GFP, C-terminal HA-Flag	(2)
Δrbp35/ Rbp35-IL (=5'UTR intron-less)	*Δrbp35*	hygr, sulph	GFP, C-terminal mRFP (cherry)	this study
***Δrbp35* (*RP27* promoter and *NOS* terminator)**				
Δrbp35/ Pr_{RP27}:*mRFP*:*RBP35*:Ter_{NOS}	*Δrbp35*	hygr, sulph	GFP, N-terminal mRFP (cherry)	this study
Δrbp35/ Pr_{RP27}:*RBP35:mRFP*:Ter_{NOS}	*Δrbp35*	hygr, sulph	GFP, C-terminal mRFP (cherry)	this study
Δrbp35/ Pr_{RP27}:*Rbp35* $_{\Delta 314\text{-}424}$:Ter_{NOS}	*Δrbp35*	hygr, sulph	GFP, C-terminal mRFP (cherry)	this study
Δrbp35/ Pr_{RP27}:*Rbp35* $_{\Delta 239\text{-}424}$:Ter_{NOS}	*Δrbp35*	hygr, sulph	GFP, C-terminal mRFP (cherry)	this study
**M35**	Guy11	hygr	T-DNA mutant (pKHt T-DNA)	(2)
M35/Rbp35-Ct (=RBP35-mRFP-C; native UTRs)	M35	hygr, sulph	C-terminal mRFP (cherry)	this study
M35/ Pr_{RP27}:*RBP35:mRFP*:Ter_{NOS}	M35	hygr, sulph	C-terminal mRFP (cherry)	this study
M35/Rbp35-IL (=5'UTR intron-less)	M35	hygr, sulph	C-terminal mRFP (cherry)	this study
Δhrp1	Guy11	hygr		this study
Δhrp1/ Hrp1:GFP	*Δhrp1*	hygr, sulph	C-terminal GFP	this study
Δhrp1/ Hrp1:HA-Flag	*Δhrp1*	hygr, sulph	C-terminal HA-Flag	this study
Δcfi25	Guy11	hygr		this study
Δcfi25/ GFP:CfI25	*ΔcfI25*	hygr,basta	N-terminal GFP	this study
Δcfi25/ HA-Flag:CfI25	*ΔcfI25*	hygr, sulph	N-terminal HA-Flag	this study

References (Table S2)

1. Leung, H., Borromeo, E., Bernardo, M. and Notteghem, J.L. (1988) Genetic analysis of virulence in the rice blast fungus *Magnaporthe grisea*. Phytopathology, 78, 1227-1233.
2. Franceschetti M, Bueno E, Wilson RA, Tucker SL, Gómez-Mena C, Calder, G. and Sesma, A. (2011) Fungal Virulence and Development Is Regulated by Alternative Pre-mRNA 3' End Processing in *Magnaporthe oryzae*. PLoS Pathogens, 7, e1002441.

Table S3. Primers used in this study.

Primer name	Sequence 5' → 3'
**General primers**	
2SKF-KpnI	AAAGGTACCAGGGAATAAGGGCGACACGGA
2SKR-KpnI	TATGGTACCTCGCCCTTCCCAACAGTTGCG
M13F	CGCCAGGGTTTTCCCAGTCACGAC
M13R	AGCGGATAACAATTTCACACAGGA
T3	AAATTAACCCTCACTAAAGGGA
**Primers for construction of RBP35:mRFP (cherry) variants**	
B3-35	GGGGACAACTTTGTATAATAAAGTTGGAAGACAAGATAAGATGAGTTGCC
B1RC-35	GGGGACTGCTTTTTTGTACAAACTTGATTCCGGGCGAGGACGTTTGGCAC
B1RNt-35	GGGGACTGCTTTTTTGTACAAACTTGTGTGTGAAAATTTGCGAGCTTGATGC
B4-RBP35	GGGGACAACTTTGTATAGAAAAGTTGGCCTTCTCATTGGTGGTTAGCCACC
B1RC-35B3a (*RBP35* $_{\Delta 239\text{-}424}$ construct)	GGGGACTGCTTTTTTGTACAAACTTGGCATGTTGTTATAACCGCCACGTCCGC
B1RC-35B1c (*RBP35* $_{\Delta 314\text{-}424}$ construct)	GGGGACTGCTTTTTTGTACAAACTTGTGCCGCCTCCCATACCACCGCGG
g145t	GTTATAACAACCGCGGACTTGGCGGTTATAACAACAT
g145t_antisense	ATGTTGTTATAACCGCCAAGTCCGCGGTTGTTATAAC
c165g	GGCGGTTATAACAACATGGGCGGCGGAATGGT
c165g_antisense	ACCATTCCGCCGCCCATGTTGTTATAACCGCC
c312g	TTGGTGGCTTCGGCGGCGGCGGA
c312g_antisense	TCCGCCGCCGCCGAAGCCACCAA
g346t	TGAACTCGAACATGCTTGGAGGTGCCATGCG
g346t_antisense	CGCATGGCACCTCCAAGCATGTTCGAGTTCA
g361t_c369g	AGGTGCCATGCTTGGAGGCGGCGGTGGTATG
g361t_c369g_antisense	CATACCACCGCCGCCTCCAAGCATGGCACCT
RevRGG3-toAGG	CCGCCGGCGAAGCCACCAAAGTTCGC
FwRGG3-toAGG	GGTGGCTTCGCCGGCGGCGGAATGGG
RevRGG1-2-toAGG	CGCCGGCCATGTTGTTATAACCGCCAGCTCCGCGGTTGTTATAACC
FwRGG1-2-toAGG	GCGGAGCTGGCGGTTATAACAACATGGCCGGCGGAATGGTC
FwRGG4-5-6-toAGG	GAACATGGCTGGAGGTGCCATGGCTGGAGGCGCCGGTGGTATGGGAG
RevRGG4-5-6-toAGG	CACCGGCGCCTCCAGCCATGGCACCTCCAGCCATGTTCGAATTCATGCC
B2R-NosTer	GGGGACAGCTTTCTTGTACAAAGTGGCTGCAGATCGTTCAAACATTTGG
B3-NosTer	GGGGACAACTTTGTATAATAAAGTTGGGGCTGCAGGAATTCTCATGTTT
**Primers for hibridisation probes**	
GSP1a	CCACGTCCGCGGTTGTTATAACCTCCG
GSP2a	GCTGTCAAGCATCGCCTGGAATCCTCAG
NGSP2	CGAGGATGGCGCTTCACAAGC
**Primers used in qRT-PCR experiments**	
Fw 03506	TTGTGAAGTCGACGAAGCAG
Rv 03506	CAGTCAGGCTTCCCCAACTA
MGG02921_qFwd	TCTCGCCTGCTGCTGTTTTGCT
MGG02921_qRev	AGCGTTGGCCTTGATGCGGT
MGG02252_qFwd	AGGTTGACGAGTATGCCGCGAG
MGG02252_qRev	TGCAAGCACCGCCGTCGATA
MGG05059_qFwd	ACGCCCACTCGGCAAACCTT
MGG05059_qRev	AAGGTCTCCCGTCCGTCCTCAAA
Fw 05283 (uricase)	GACAATGTCCGCGTCTACAA
Rv 05283 (uricase)	AGGCAGCATACGGTCATCTC
Fw 10088	CTCCCATGGCACATGAAAC
Rv 10088	GGAGTGTCGGTAGCTCGGTA
Fw 08359 (flavonol syntase)	GGCTACCTCAAGAGCAGCAT
Rv 08359 (flavonol syntase)	CAAAGTAGAGCAGCCCCAGA
Fw 07066 (Ca-binding mit. carrier)	GCGGTCAAGGAGACATTGAT
Rv 07066 (Ca-binding mit. carrier)	CACTGACGGCATACTTGGTG
Rv-09757gluc	GCAGGTAGACCTTGGAGCTG
Fw-09757gluc	CGGTGAGACCCAGTACCTGT
MGG09075_qFwd	TTCGGACAAGTTCCGCGGCT
MGG09075_qRev	TCCTCAACTGCACTCCGTGCCA

MGG_10583_F	GAACCGTGGCACGCCGGTCA
MGG_10583_R	GCGTGCACGGCCTCGGTCAC
MGG_08201_F	GCGATGATTGCCAGCGCCAGA
MGG_08201_R	GCAATGGGCGAGCCGCACTC
MGG_04337_F	GGGACCCCGAAGCCGCTGGT
MGG_04337_R	TGCGTGCACCGTTGGCTCCAC
actin Fw	CCTGGCACCGTCGTCGATGAAGG
actin Rv	GCGAGGCGAGAATGGAACCAC
qPCR-RBP35F2	GGCTGCGAGGAGGAGTTGAAGG
qPCR-RBP35R2	CCGCCTGCTGGGATGTGAGC
HA-qFor	CTACGCCGGCTACCCCTACG
HA-qRev	ATCGTCGTCCTTGTAGTCGCTGCAC

Delta and tagging of CFI25 and HRP1

B4-LB-hrp1deltaF	GGGGACAACTTTGTATAGAAAAGTTGGGAGCCATATAGCTGAGGCCAC
B1R-LB-hrp1deltaR	GGGGACTGCTTTTTTGTACAAACTTGCCTTACGATGAACCGGAGTGGT
B2R-RB-hrp1deltaF	GGGGACAGCTTTCTTGTACAAAGTGGAGGACGACTGAGAGGCAAACTG
B3-RB-hrp1deltaR	GGGGACAACTTTGTATAATAAAGTTGGTAAGCTGACCGCATCTGATGGC
B1RC-hrp1	GGGACTGCTTTTTTGTACAAACTTGAGCCGCGGGCATATGGATGGAAG
B4-CFIm	GGGGACAACTTTGTATAGAAAAGTTGCCCGAAGGGTTCCCAATCGCTTC
B1RNt-CFIm	GGGGACTGCTTTTTTGTACAAACTTGTGTGAAAACGAGATGCCGATCCG
B3-CFlm	GGGGACAACTTTGTATAATAAAGTTGCGTGAGGGAGCATATGAGCAATGC
B2R-CFIm	GGGGACAGCTTTCTTGTACAAAGTGGTCATGTCAACCATCACAACAAC
B2RforKO-CFI	GGGGACAGCTTTCTTGTACAAAGTGGACCATAGAGGTCCTGAGCGTCCC
B1-Hygr	GGGGACAAGTTTGTACAAAAAAGCAGGCTCCGTCGACGTTAACTGATATTG
B2-Hygr-stop	GGGGACCACTTTGTACAAGAAAGCTGGGTACTATTCCTTTGCCCTCGGAC

Confirmation Delta strains

hrp 5 For	GGAACTCAACAGAAGATGC
HRP1 3' out Rev	GCTGCACAAGAGCGAGAAGAACCTG
CFI25 5 for	GAGGTAAAACTCCCTTACC
CF25 3' out Rev	GGCTTGCTCAACTTGTTTGTCCAGC

Confirmation by RT-PCR of M35 fusion transcripts

RBP35-fusionM35For	GTTGATCGGCACGTAAGAGG
RBP35-fusionM35For1	AGGCAGTTATTGGTGCCCTT
RBP35-fusionM35Rev	CTTCCTGCCTGCTTGTGAAG
RBP35-fusionM35Rev1	AGTCCAGATCCCTATTCCGG

Cloning by RT-PCR the 3' ends of *uORF1* and *uORF2* transcripts

µORF-For	acgtATGGTCAAAGTTCCGC
µORFa-Rev	TTTTTTTTTTACATGAAGATTATGAAAAGA
µORFb-Rev	TTTTTTTTTTAGCTGTATTGAAAAGGCCTA
